# Supplementary material for: Effects of digital health counseling and behavioral interventions on weight management during pregnancy and postpartum: A systematic review and meta-analysis of randomized controlled trials
Source: PLoS One. 2025 Sep 25;20(9):e0331913. doi: 10.1371/journal.pone.0331913 (PMC12463243; doi:10.1371/journal.pone.0331913)
Supplement: S1 Appendix — (DOCX) [file pone.0331913.s001.docx]

| **S1 Appendix**. Search strategy for MEDLINE, Embase, PsychInfo, and Proquest. | | |
| --- | --- | --- |
| **Database** | **Search Concept** | **Search Terms** |
| **MEDLINE (OVID)** | **Population** | exp Pregnancy; exp Postpartum; exp pregnant women; exp Prenatal Care; exp perinatal care; exp postnatal care; (pregnan* or gestation* or matern* or antenatal or ante-natal or prenatal or pre-natal or perinatal or peri-natal or postpartum or post-partum postnatal post-natal peripartum peri partum).mp. |
|  | **Digital Intervention** | exp Cell Phone; exp Internet; exp Computers, Handheld; exp computers; exp software; exp Computer-Assisted Instruction; Wireless Technology; electronic mail/; Telemedicine/; Mobile Applications; telenursing; app or apps or mobile app* or ehealth or e-health or mhealth or mobile health or m-health or telehealth or tele-health or internet or web or technology or smartphone or smart phone or smart-phone or cell-phone or cell phone or cellular phone or online or on-line or "text adj messag*" or SMS or "world wide web" or digital or mobile or computer-based or website or tablet or PDA or "personal digital assistant" or messaging or email or android or iphone or ipad or Computer-Assisted |
|  | **Counseling & Behavioral** | exp Exercise; exp physical fitness; counseling/ or exp directive counseling/; exp Behavior Therapy/; exp Motivation/; health behavior/ or risk reduction behavior/; exp Patient Education as Topic/; exp social support/; health education/ or exp health promotion/; exp Diet; exp Nutrition Therapy; Exp Life Style; Exp Body Weight; weight manag* or weight control or fitness or "healthy eating"; (diet* or nutrition or "physical activity" or "physical exertion" or exerci* or weight).ti,ab.; (lifestyle or "life style" or advice or advise or behavior* or behaviour* or nonpharma* or "non-pharma*" or activ* fitness).ti,ab. |
| **EMBASE** | **Population** | exp pregnancy/; postnatal care/; prenatal care/; perinatal care/ or maternal care/; (pregnant or pregnancy).tw.; (postpartum or post partum or post natal or postnatal or antenatal or ante-natal or prenatal or prenatal or perinatal or peri-natal or pregnan* or gestation* or matern*).tw. |
|  | **Digital Intervention** | internet or telemedicine/; exp microcomputer/ or exp mobile phone/ or exp Internet/ or exp personal digital assistant/; e-mail/ or social media/ or text messaging/ or videoconferencing/ or webcast/ or wireless communication/; ("cellular phone" or cell phone? or mobile or internet or web or computer-based or website or tablet or PDA or messaging or online).ti,ab.; (blog or twitter or snapchat or social media or instragram or facebook or ehealth).tw.; (smartphone or smart phone or android or iphone or ipad).ti,ab. |
|  | **Counseling & Behavioral** | Physical activity; Exercise; Lifestyle; Healthy Lifestyle; Diet; Diet Therapy; Body Weight Gain; Body Weight Control; counseling/ or exp directive counseling/; exp Behavior Therapy/; exp Motivation/; health behavior/ or risk reduction behavior/; exp Patient Education as Topic/; exp social support/; health education/ or exp health promotion/ |
| **Cochrane Library** | **Population** | exp pregnancy/; postnatal care/; prenatal care/; perinatal care/ or maternal care/; (pregnant or pregnancy).tw.; (postpartum or post partum or post natal or postnatal or antenatal or ante-natal or prenatal or prenatal or perinatal or peri-natal or pregnan* or gestation* or matern*).tw. |
|  | **Digital Intervention** | internet or telemedicine/; exp microcomputer/ or exp mobile phone/ or exp Internet/ or exp personal digital assistant/; e-mail/ or social media/ or text messaging/ or videoconferencing/ or webcast/ or wireless communication/; ("cellular phone" or cell phone? or mobile or internet or web or computer-based or website or tablet or PDA or messaging or online).ti,ab.; (blog or twitter or snapchat or social media or instragram or facebook or ehealth).tw.; (smartphone or smart phone or android or iphone or ipad).ti,ab. |
|  | **Counseling & Behavioral** | Physical activity; Exercise; Lifestyle; Healthy Lifestyle; Diet; Diet Therapy; Body Weight Gain; Body Weight Control; counseling/ or exp directive counseling/; exp Behavior Therapy/; exp Motivation/; health behavior/ or risk reduction behavior/; exp Patient Education as Topic/; exp social support/; health education/ or exp health promotion/ |
| **PsychInfo** | **Population** | exp pregnancy/; postnatal care/; prenatal care/; perinatal care/ or maternal care/; (pregnant or pregnancy).tw.; (postpartum or post partum or post natal or postnatal or antenatal or ante-natal or prenatal or prenatal or perinatal or peri-natal or pregnan* or gestation* or matern*).tw. |
|  | **Digital Intervention** | internet or telemedicine/; exp microcomputer/ or exp mobile phone/ or exp Internet/ or exp personal digital assistant/; e-mail/ or social media/ or text messaging/ or videoconferencing/ or webcast/ or wireless communication/; ("cellular phone" or cell phone? or mobile or internet or web or computer-based or website or tablet or PDA or messaging or online).ti,ab.; (blog or twitter or snapchat or social media or instragram or facebook or ehealth).tw.; (smartphone or smart phone or android or iphone or ipad).ti,ab. |
|  | **Counseling & Behavioral** | Physical activity; Exercise; Lifestyle; Healthy Lifestyle; Diet; Diet Therapy; Body Weight Gain; Body Weight Control; counseling/ or exp directive counseling/; exp Behavior Therapy/; exp Motivation/; health behavior/ or risk reduction behavior/; exp Patient Education as Topic/; exp social support/; health education/ or exp health promotion/ |
| **ProQuest** | **Population** | MESH.EXACT.EXPLODE("Postnatal Care") OR MESH.EXACT.EXPLODE("Prenatal Care") OR MESH.EXACT.EXPLODE("Perinatal Care") OR MESH.EXACT.EXPLODE("Pregnancy") OR ABSTRACT,TITLE(antenatal OR antepartum OR prenatal OR prenatal OR perinatal OR peripartum OR postnatal OR postnatal OR postpartum OR pregnancy OR pregnant) |
|  | **Digital Intervention** | MESH.EXACT.EXPLODE("Therapy, Computer-Assisted") OR MESH.EXACT.EXPLODE("Internet") OR MESH.EXACT.EXPLODE("Computers") OR MESH.EXACT.EXPLODE("text messaging") OR ABSTRACT,TITLE(phone* OR computer-assisted OR computer-based OR web-based OR Telemedicine OR "communication network" OR "remote consultation" OR "electronic health" OR "mobile health" OR telehealth OR e-health OR m-health OR computer* OR internet OR online OR "world wide web" OR website* OR telecare OR telemonitoring OR interactive OR mobile OR "personal digital assistant" OR PDA OR "text message" OR "text messaging" OR "text message" OR SMS OR "instant message" OR "social network" OR tailored OR remote OR self-monitoring) |
|  | **Counseling & Behavioral** | MESH.EXACT.EXPLODE("Obesity") OR MESH.EXACT.EXPLODE("Weight Gain") OR MESH.EXACT.EXPLODE("Body Mass Index") OR MESH.EXACT.EXPLODE("Overweight") OR MESH.EXACT.EXPLODE("Weight Loss") OR MESH.EXACT.EXPLODE(Diet) OR MESH.EXACT.EXPLODE("Physical Fitness") OR MESH.EXACT.EXPLODE("Body Weight") OR MESH.EXACT.EXPLODE(Counseling) OR "Behavior modification" OR ABSTRACT,TITLE (overweight OR obese* OR BMI OR weight OR exercise OR sport* OR exercise OR "physical exertion" OR physical exercise* OR "physical activity" OR diet* OR "healthy eating" OR nutrition OR lifestyle OR "life style" OR "weight gain" OR "weight manag*" OR "weight control" OR counseling) |
|  | **Study Design** | placebo OR trial OR random* control* OR "control* trial" OR pilot OR feasibility |
