# Supplementary material for: Effects of digital health counseling and behavioral interventions on weight management during pregnancy and postpartum: A systematic review and meta-analysis of randomized controlled trials
Source: PLoS One. 2025 Sep 25;20(9):e0331913. doi: 10.1371/journal.pone.0331913 (PMC12463243; doi:10.1371/journal.pone.0331913)
Supplement: S2 Appendix — (DOCX) [file pone.0331913.s002.docx]

**S2 Appendix.** Risk of bias assessment.

*
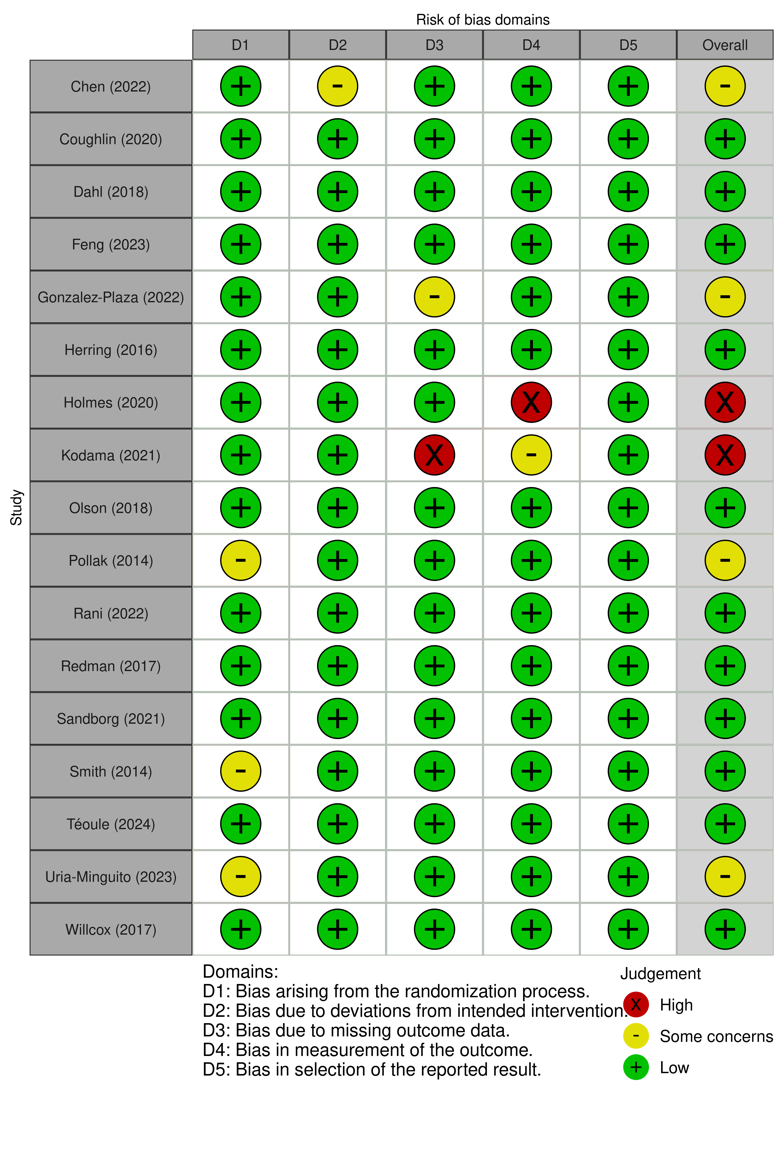
*

**Figure A*.*** Risk of bias for studies reporting gestational weight gain (in kilogram) for digital health vs. usual care.

*
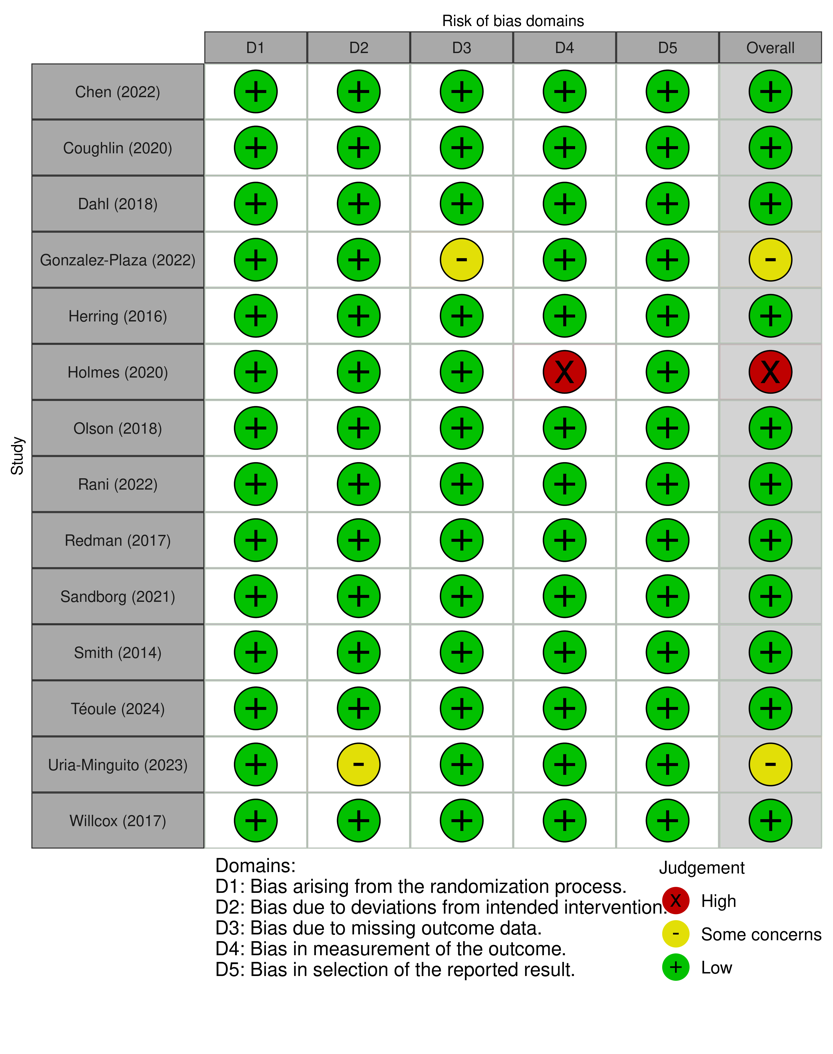
*

**Figure B*.*** Risk of bias for studies reporting gestational weight gain exceeding Institute of Medicine (IOM) recommendations for digital health vs. usual care.

*
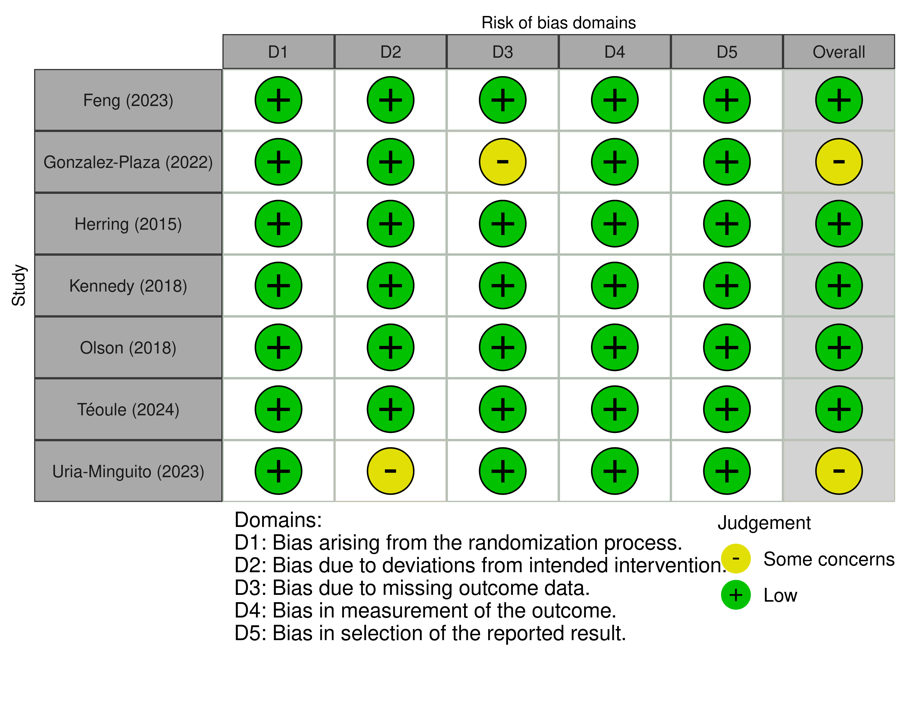
*

**Figure C***.* Risk of bias for studies reporting gestational diabetes mellitus for digital health vs. usual care.

*
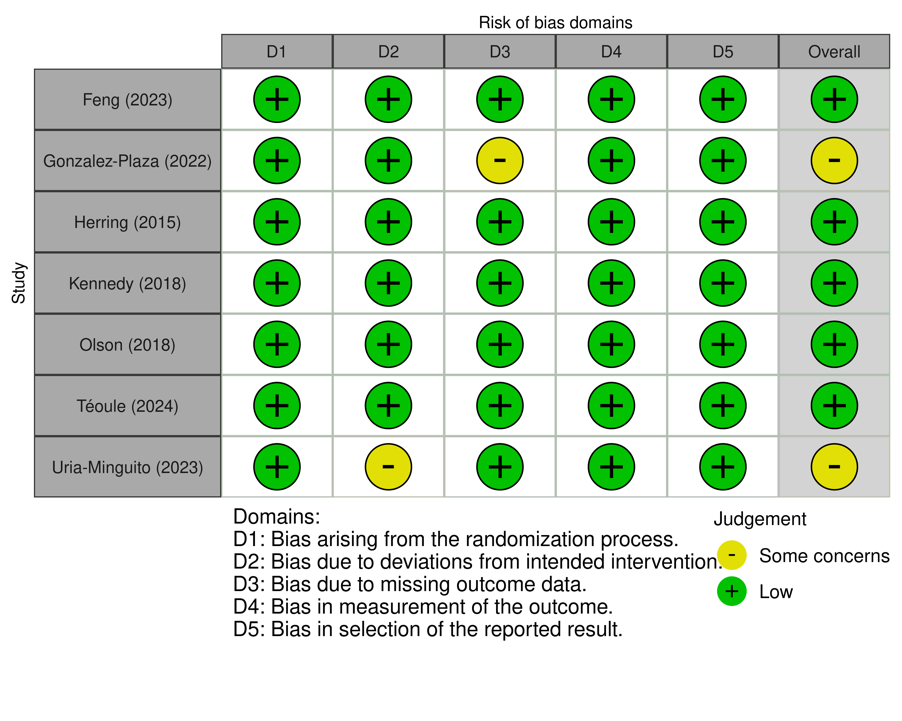
*

**Figure D*.*** Risk of bias for studies reporting C-section for digital health vs. usual care.

*
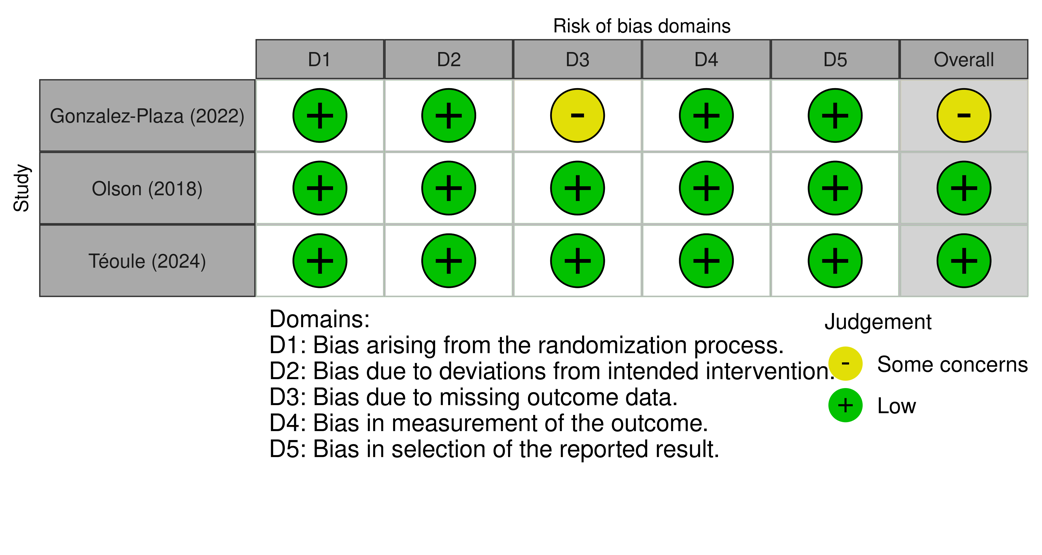
*

**Figure E*.*** Risk of bias for studies reporting pre-eclampsia for digital health vs. usual care.

*
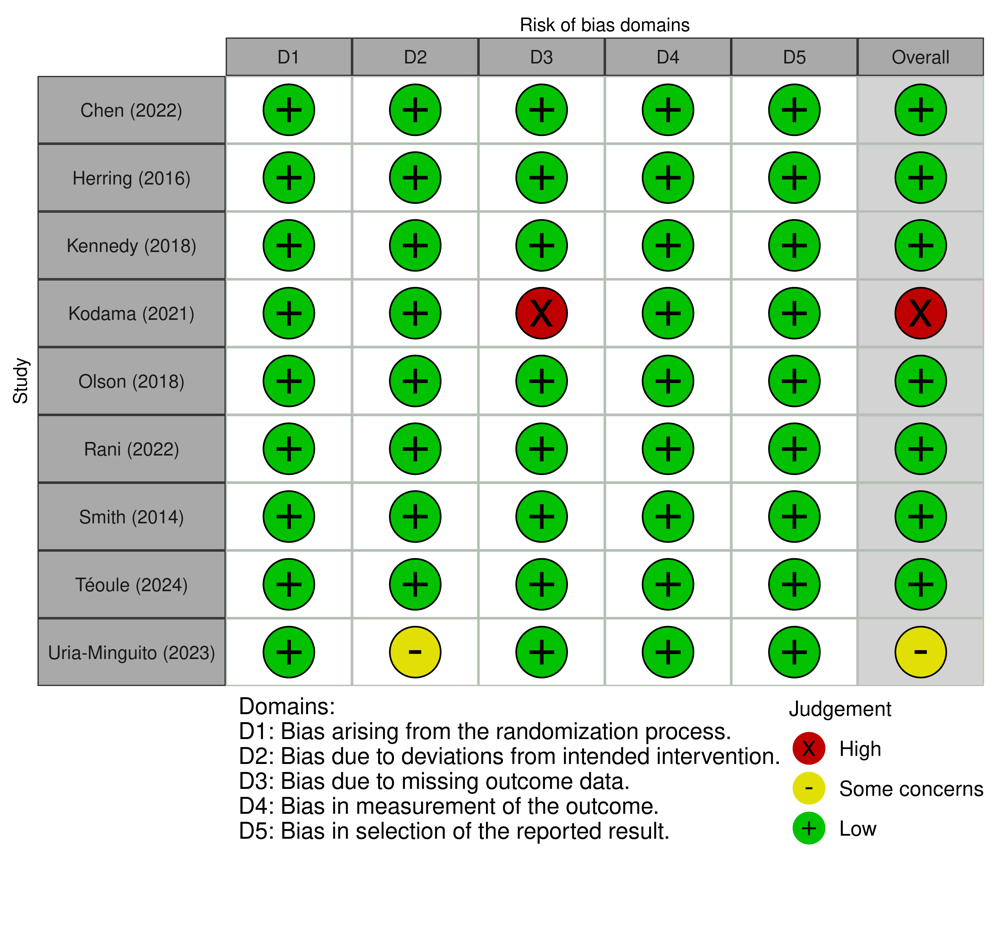
*

**Figure F*.*** Risk of bias for studies reporting birth weight for digital health vs. usual care.

*
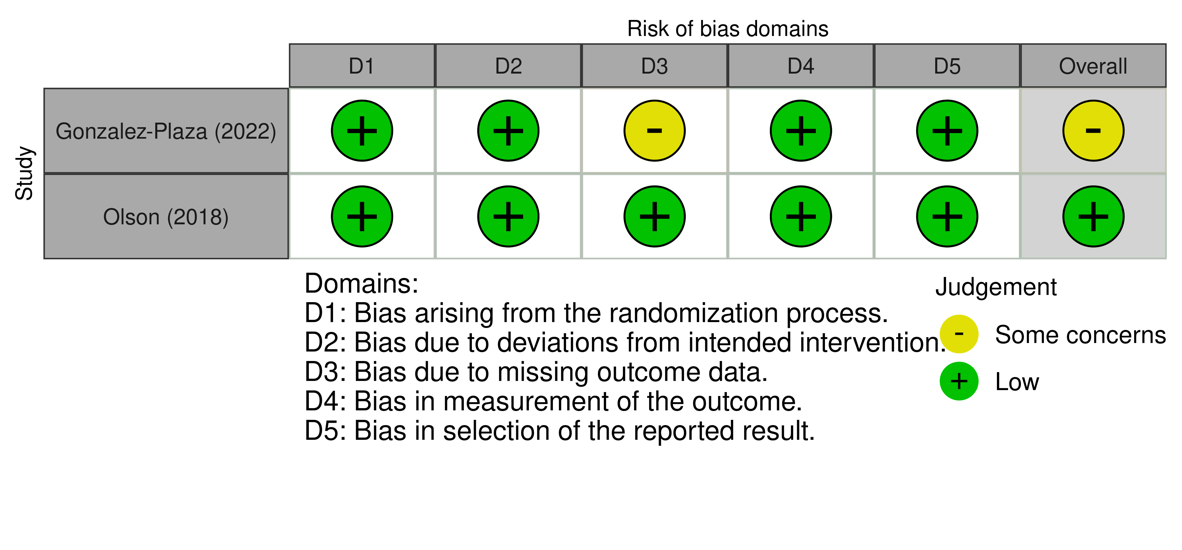
*

**Figure G*.*** Risk of bias for studies reporting preterm birth for digital health vs. usual care.
