# Supplementary material for: Effects of digital health counseling and behavioral interventions on weight management during pregnancy and postpartum: A systematic review and meta-analysis of randomized controlled trials
Source: PLoS One. 2025 Sep 25;20(9):e0331913. doi: 10.1371/journal.pone.0331913 (PMC12463243; doi:10.1371/journal.pone.0331913)
Supplement: S3 Appendix — (DOCX) [file pone.0331913.s003.docx]

**S3 Appendix.** Forest plots.


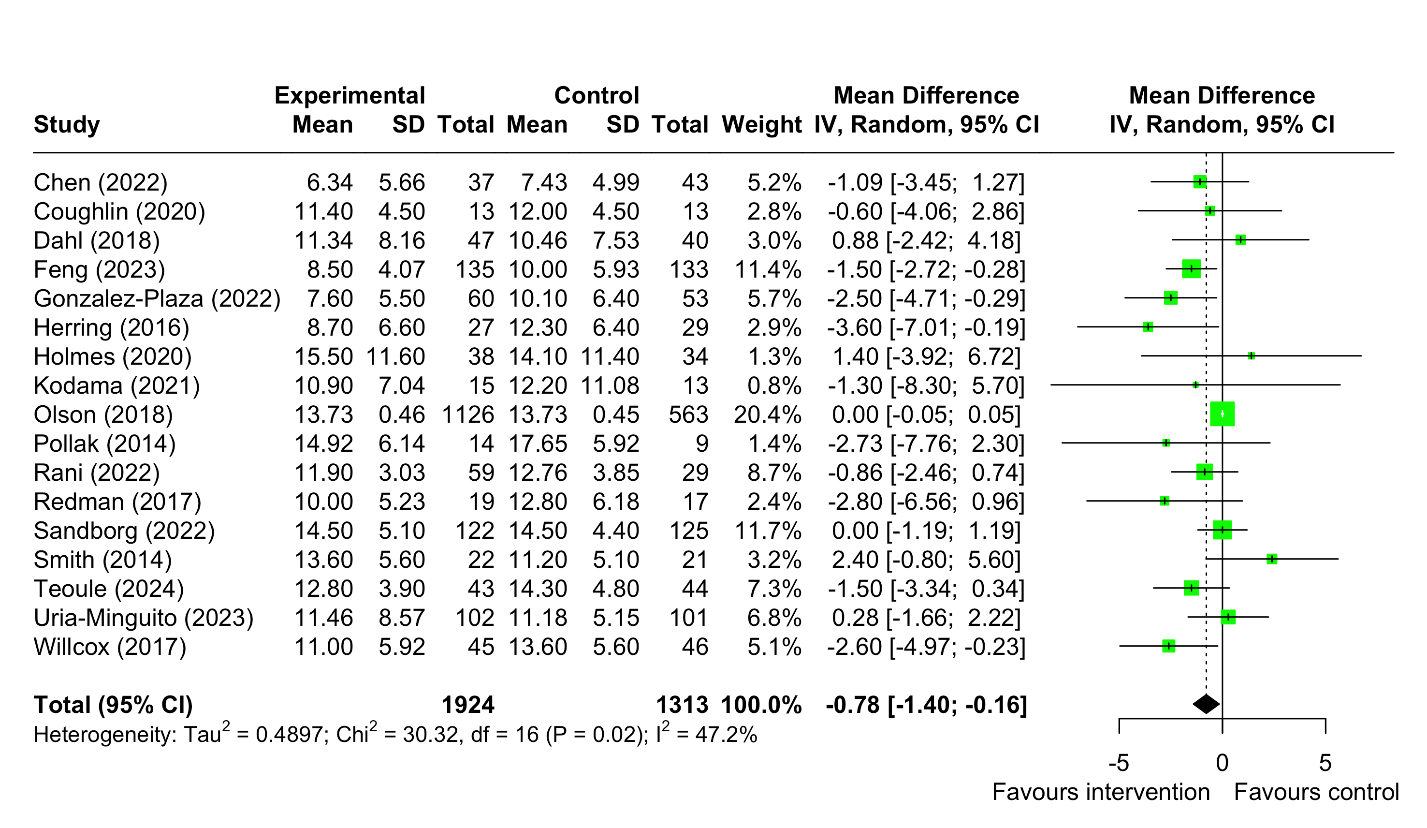


**Figure A*.*** Mean difference for gestational weight gain (in kilogram) for digital health vs. usual care. The pooled effect is calculated by the DerSimonian-Laird random effects model.


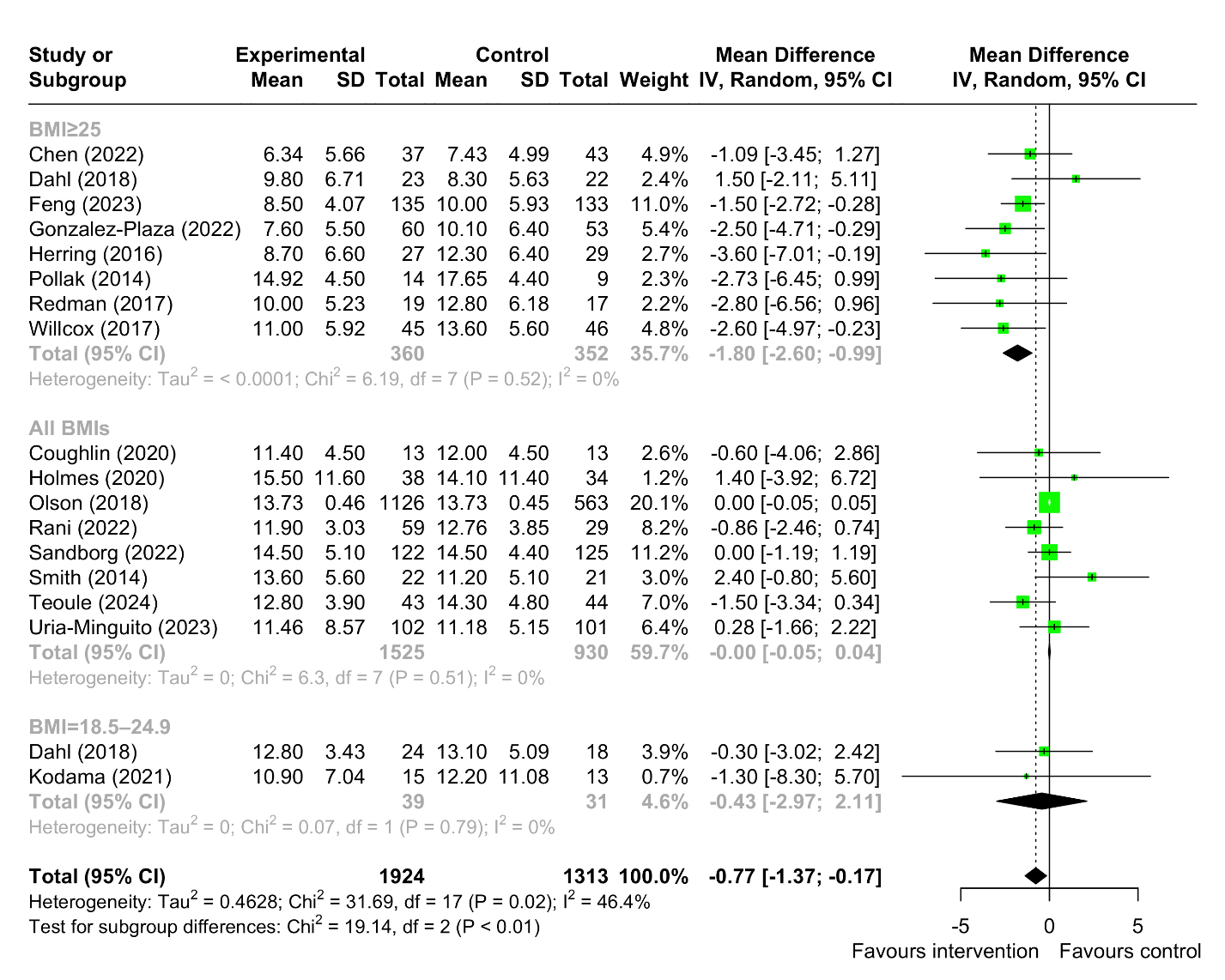


**Figure B*.*** Mean difference for gestational weight gain (in kilogram) for digital health vs. usual care. The pooled effect is calculated by the DerSimonian-Laird random effects model. The subgroup is based on body mass index (BMI) categories.


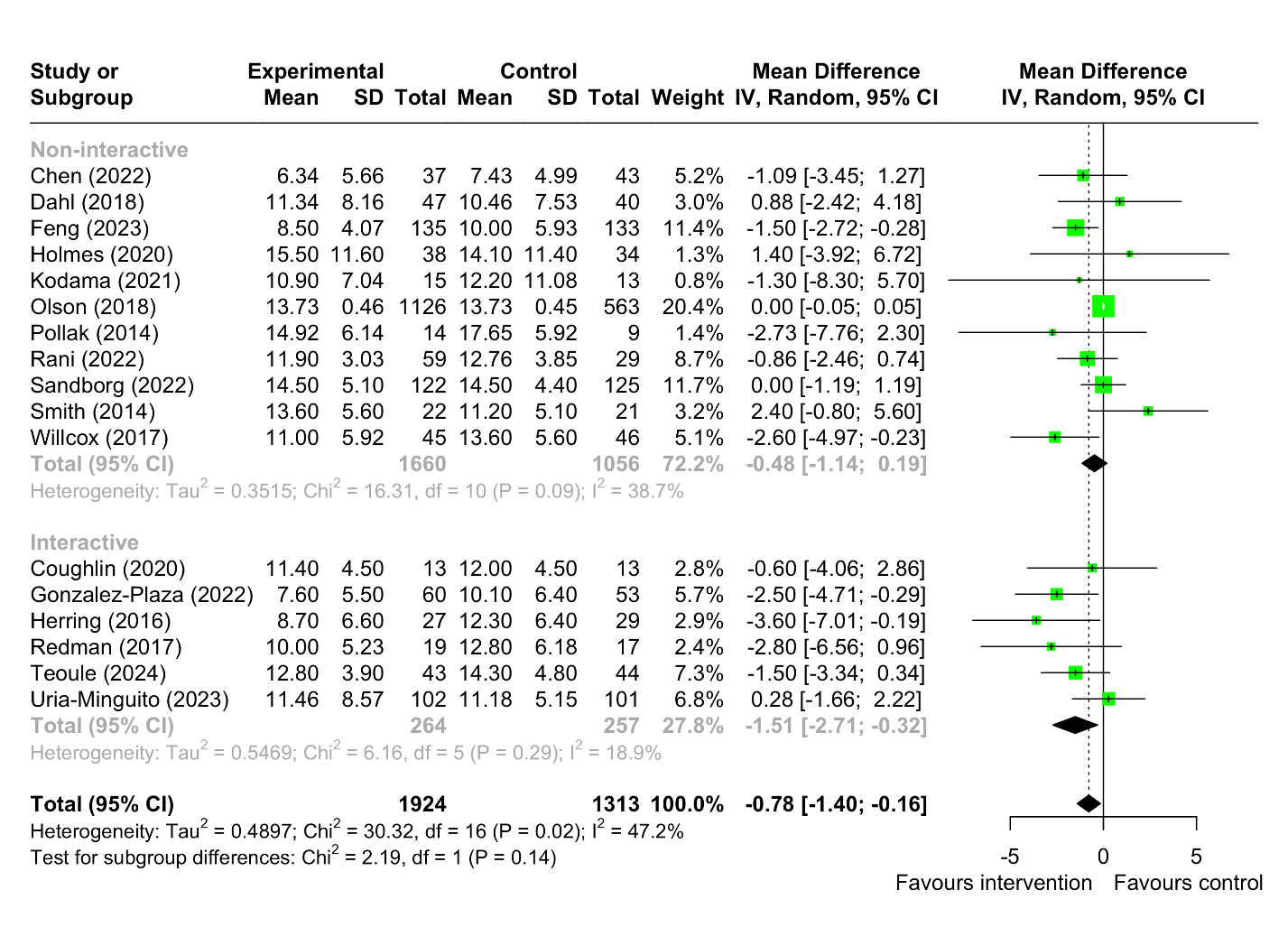


**Figure C*.*** Mean difference for gestational weight gain (in kilogram) for digital health vs. usual care. The pooled effect is calculated by the DerSimonian-Laird random effects model. The subgroup is based on the interactivity of the digital health intervention.


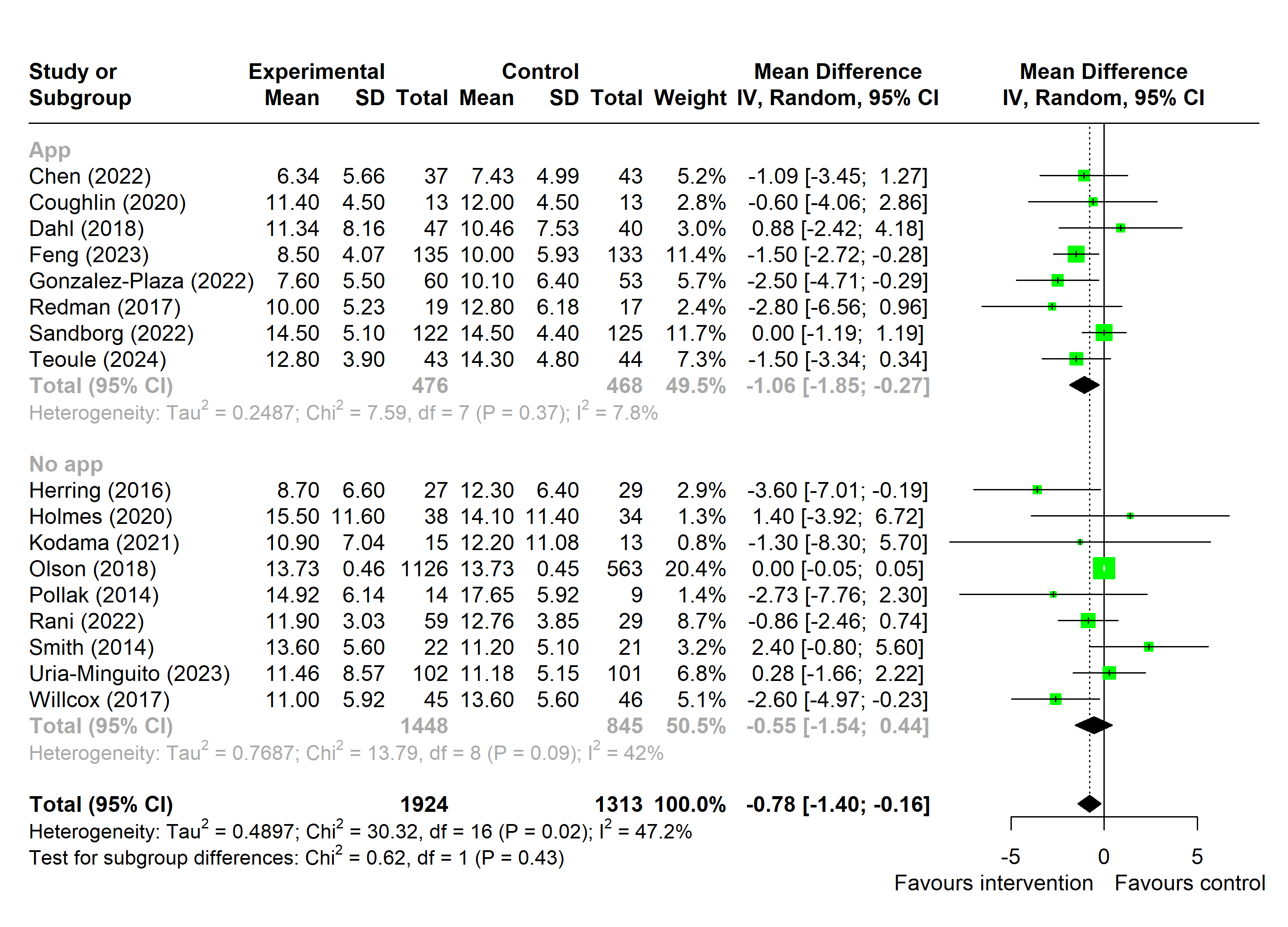


**Figure D*.*** Mean difference for gestational weight gain (in kilogram) for digital health vs. usual care. The pooled effect is calculated by the DerSimonian-Laird random effects model. The subgroup is based on incorporating a mobile app in the digital health intervention.


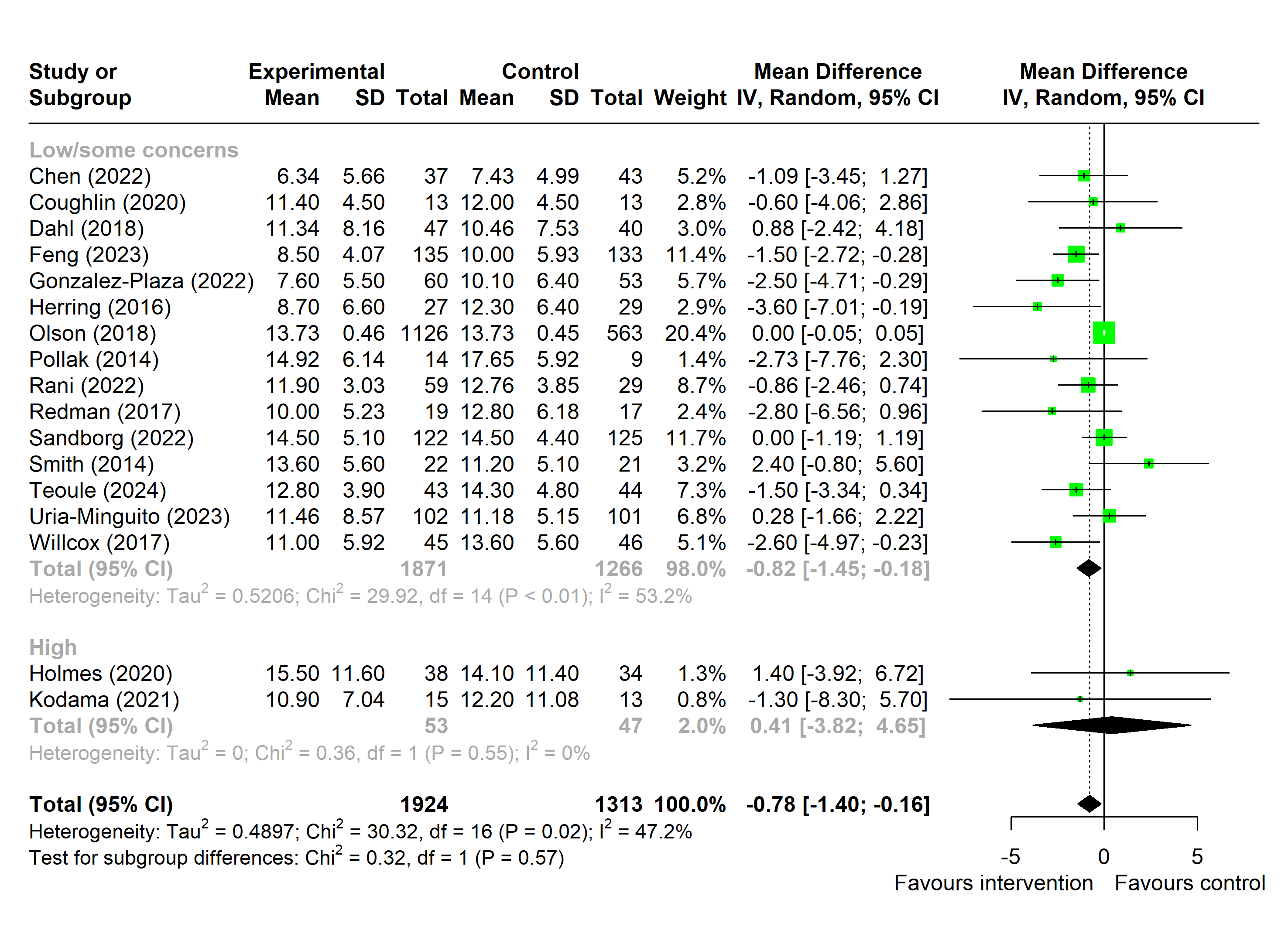


**Figure E*.*** Mean difference for gestational weight gain (in kilogram) for digital health vs. usual care. The pooled effect is calculated by the DerSimonian-Laird random effects model. The subgroup is based on the overall risk of bias of the study.


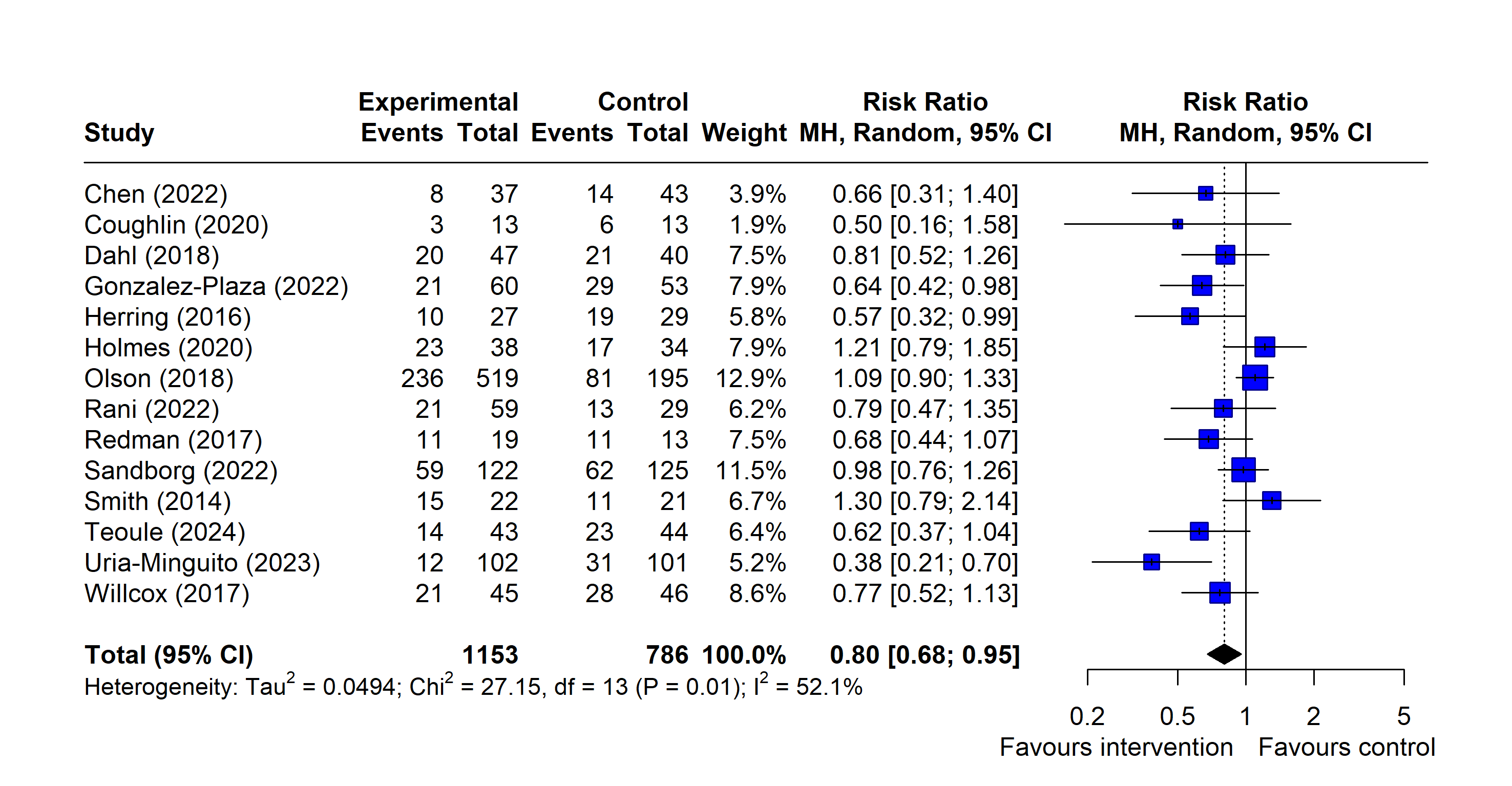


**Figure F*.***  Risk ratio for gestational weight gain exceeding Institute of Medicine (IOM) recommendations for digital health vs. usual care. The pooled effect is calculated by the Mantel–Haenszel random effects model.


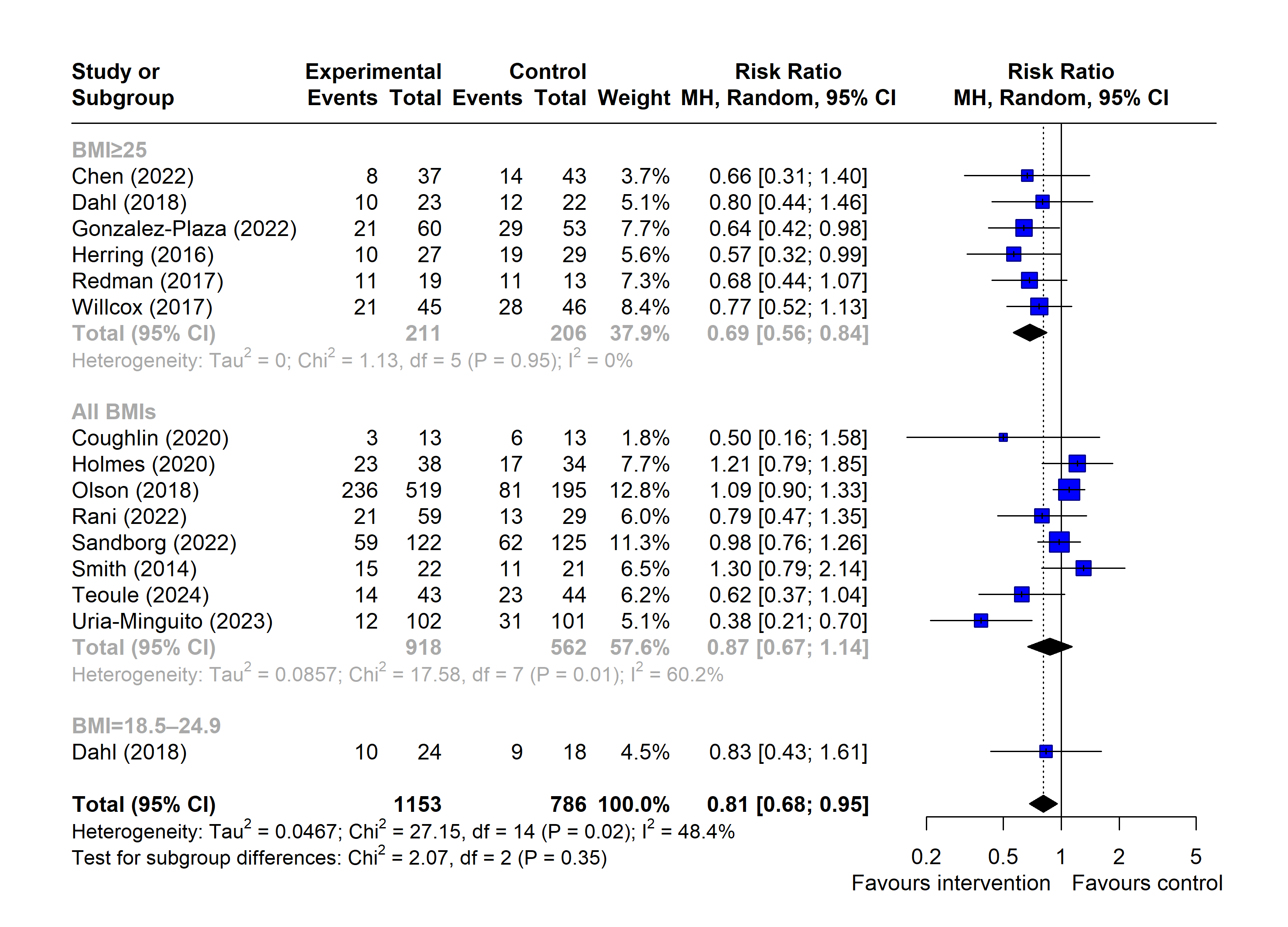


**Figure G*.*** Risk ratio for gestational weight gain exceeding Institute of Medicine (IOM) recommendations for digital health vs. usual care. The pooled effect is calculated by the Mantel–Haenszel random effects model. The subgroup is based on body mass index (BMI) categories.


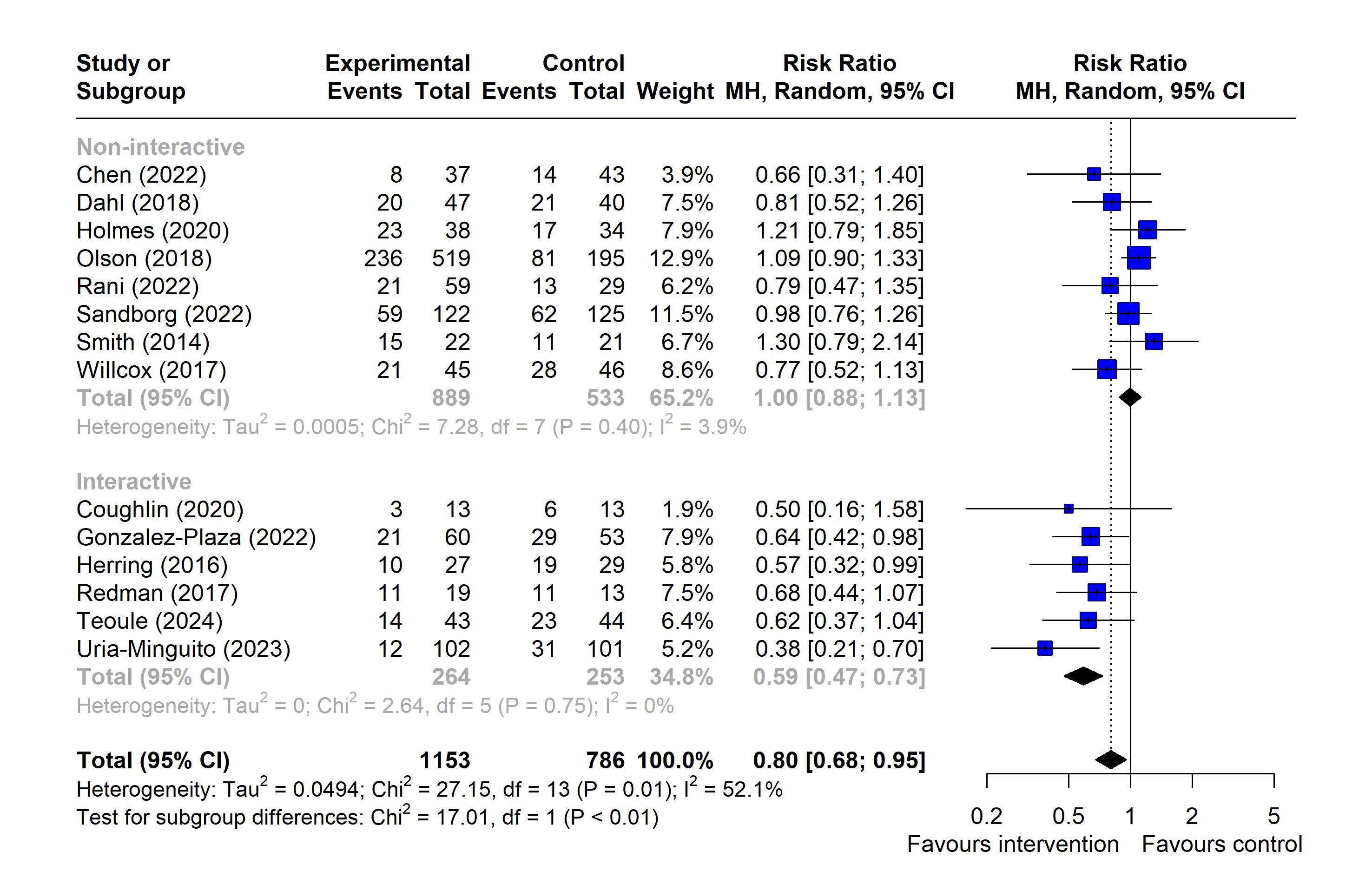


**Figure H*.*** Risk ratio for gestational weight gain exceeding Institute of Medicine (IOM) recommendations for digital health vs. usual care. The pooled effect is calculated by the Mantel–Haenszel random effects model. The subgroup is based on the interactivity of the digital health intervention.

*
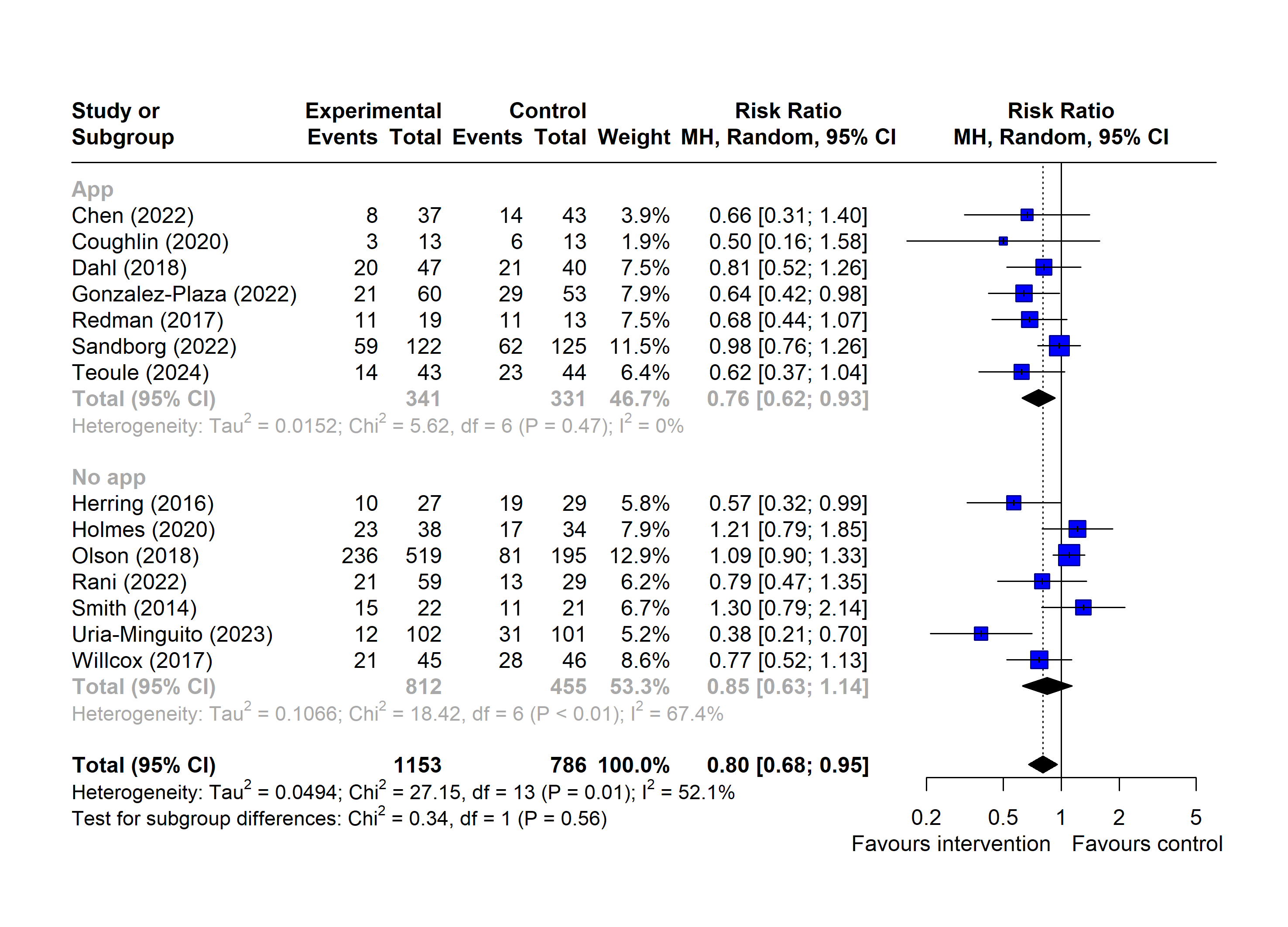
*

**Figure I*.*** Risk ratio for gestational weight gain exceeding Institute of Medicine (IOM) recommendations for digital health vs. usual care. The pooled effect is calculated by the Mantel–Haenszel random effects model. The subgroup is based on incorporating a mobile app in the digital health intervention.


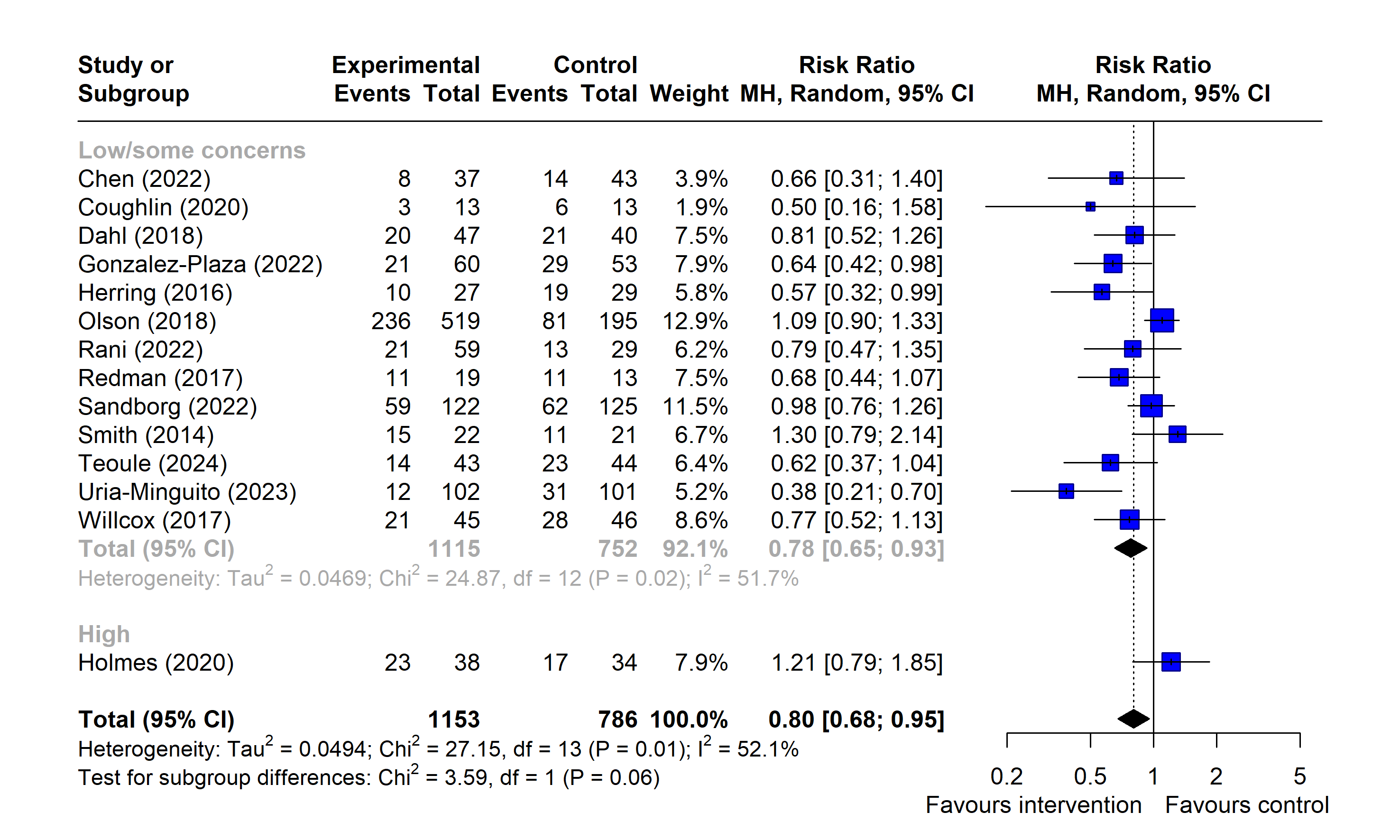


**Figure J*.*** Risk ratio for gestational weight gain exceeding Institute of Medicine (IOM) recommendations for digital health vs. usual care. The pooled effect is calculated by the Mantel–Haenszel random effects model. The subgroup is based on the overall risk of bias of the study.


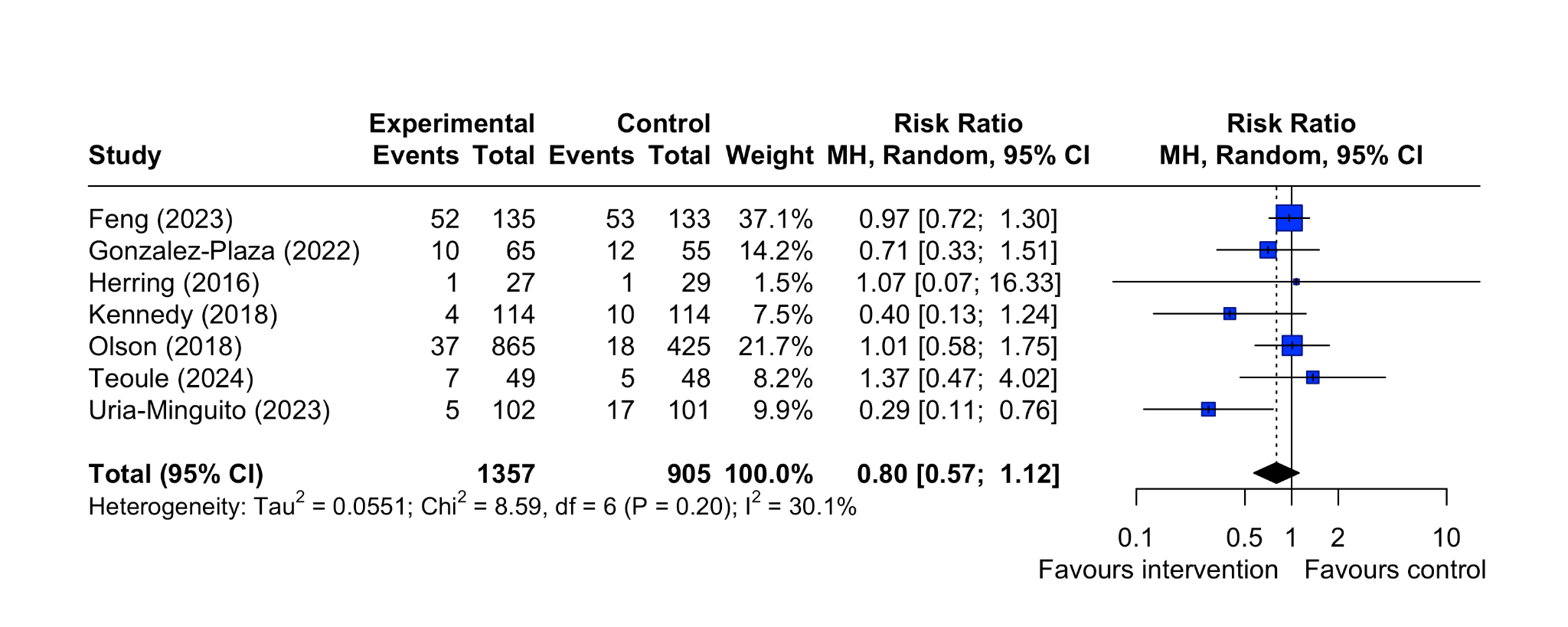


**Figure K*.*** Risk ratio for gestational diabetes mellitus for digital health vs. usual care. The pooled effect is calculated by the Mantel–Haenszel random effects model.


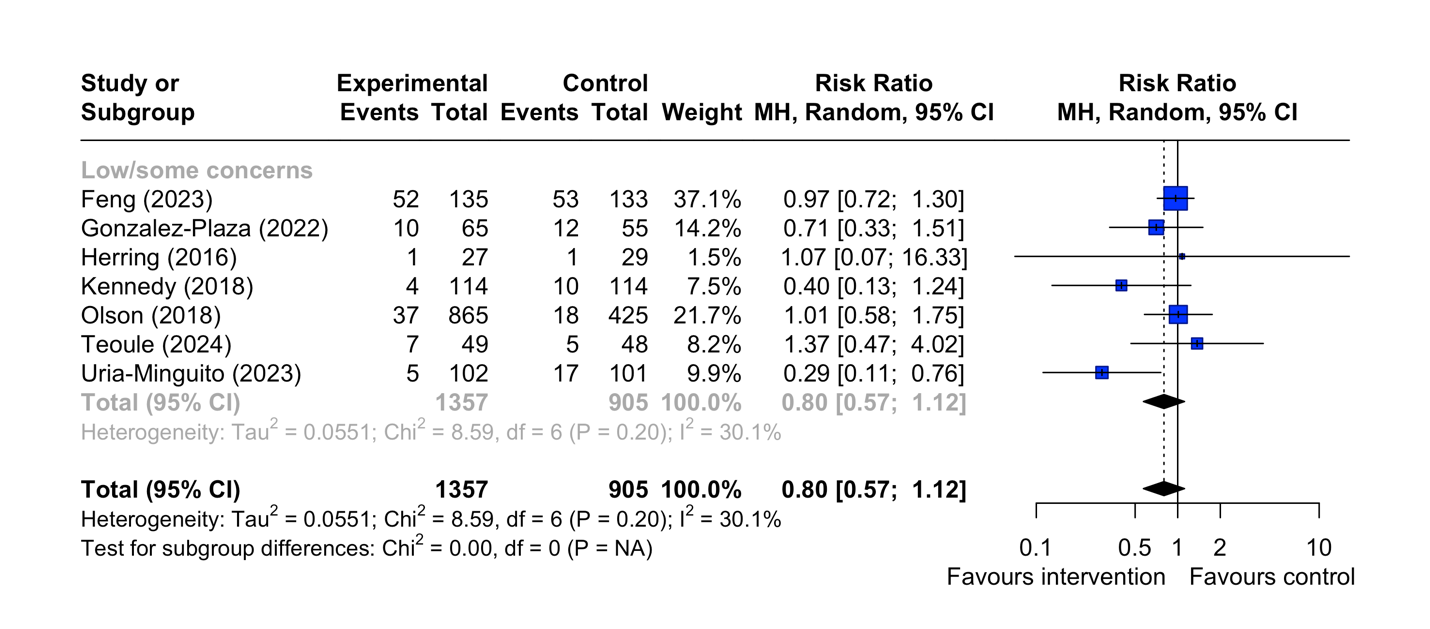


**Figure L*.*** Risk ratio for gestational diabetes mellitus for digital health vs. usual care. The pooled effect is calculated by the Mantel–Haenszel random effects model. The subgroup is based on the overall risk of bias of the study.


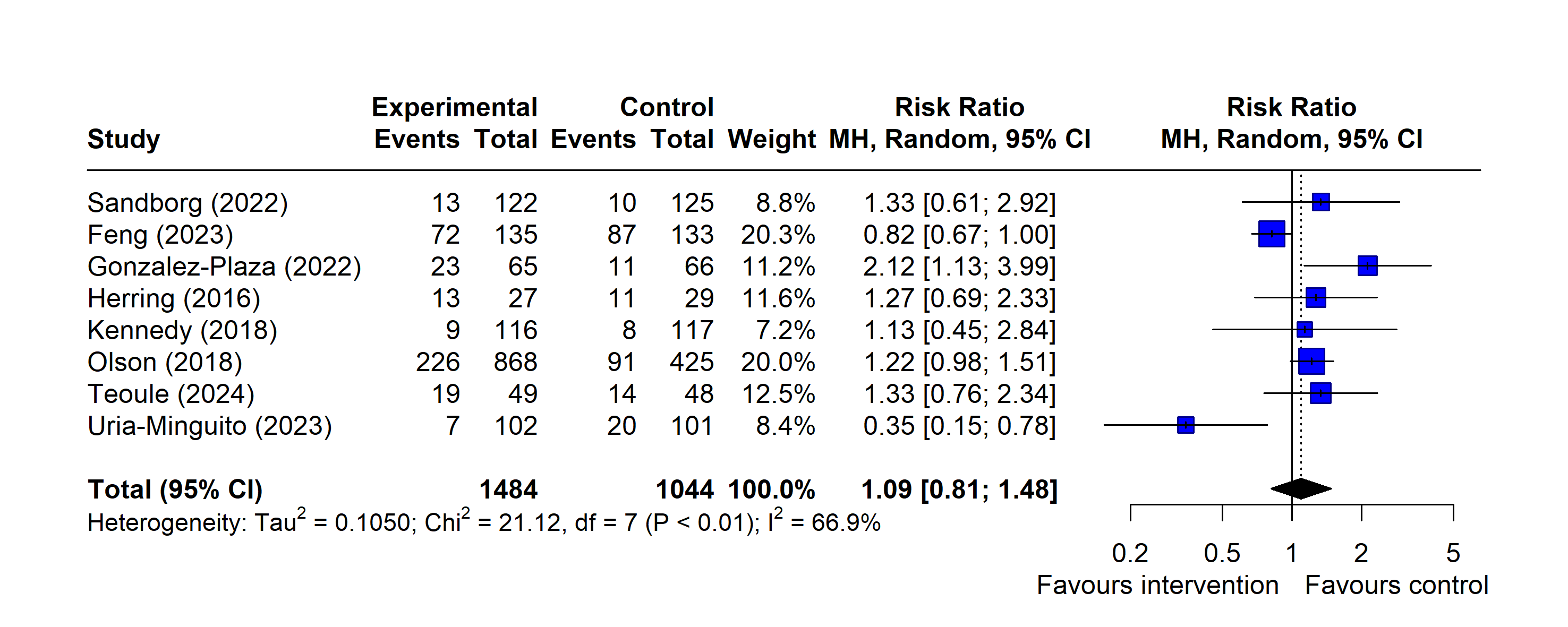


**Figure M*.*** Risk ratio for cesarean birth for digital health vs. usual care. The pooled effect is calculated by the Mantel–Haenszel random effects model.


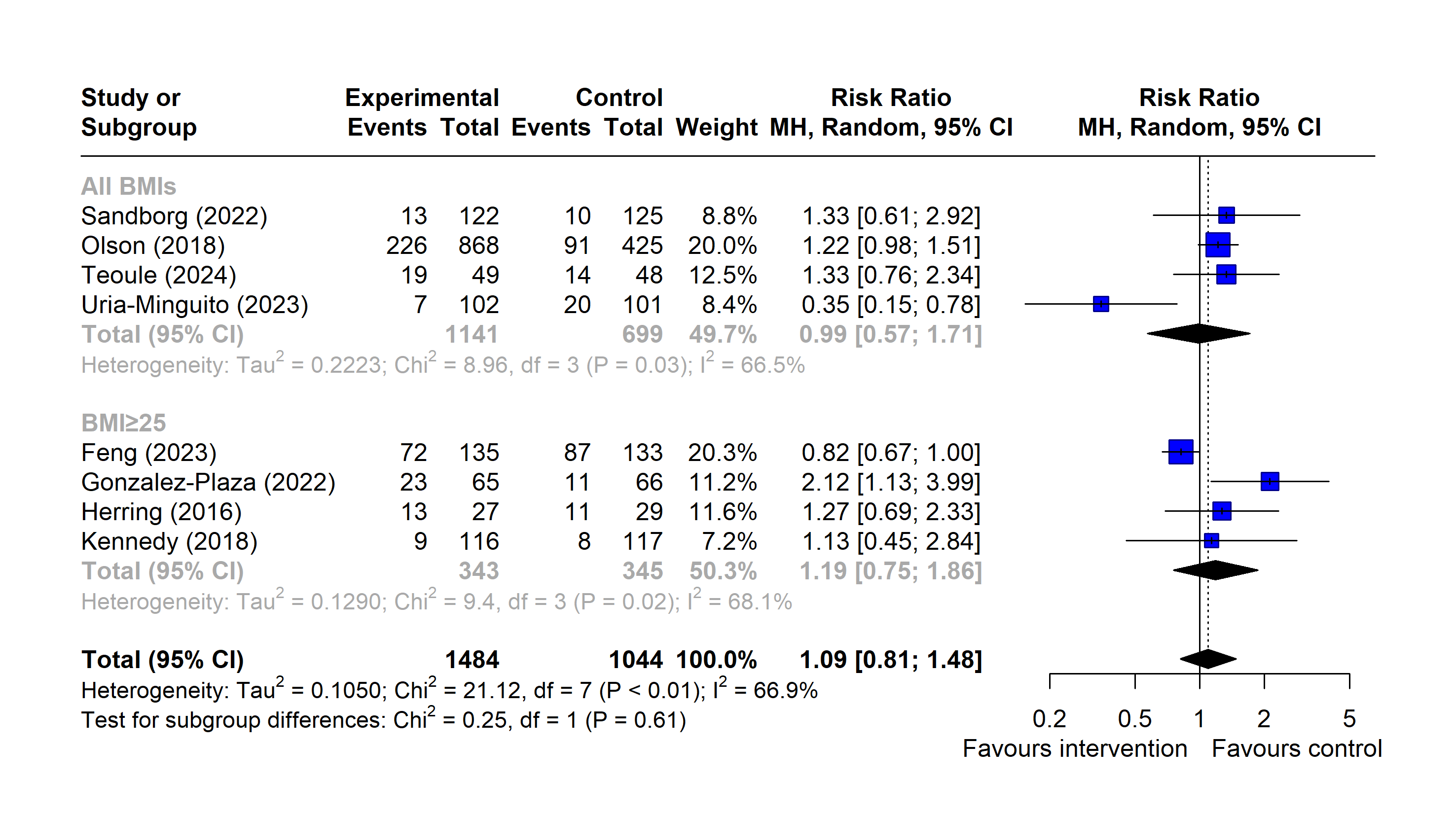


**Figure N*.*** Risk ratio for cesarean birth for digital health vs. usual care. The pooled effect is calculated by the Mantel–Haenszel random effects model. The subgroup is based on body mass index (BMI) categories.


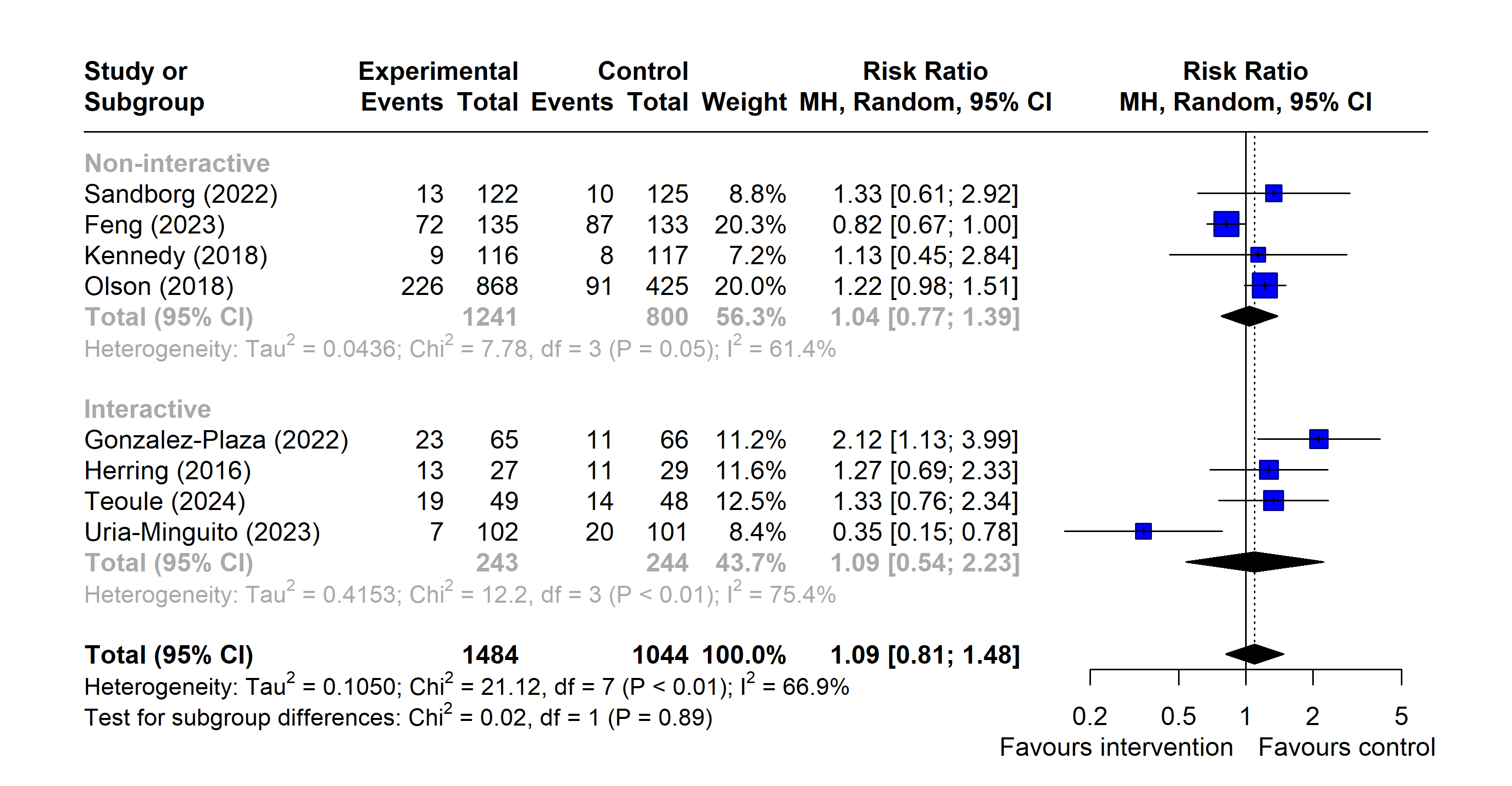


**Figure O*.*** Risk ratio for cesarean birth for digital health vs. usual care. The pooled effect is calculated by the Mantel–Haenszel random effects model. The subgroup is based on the interactivity of the digital health intervention.

*
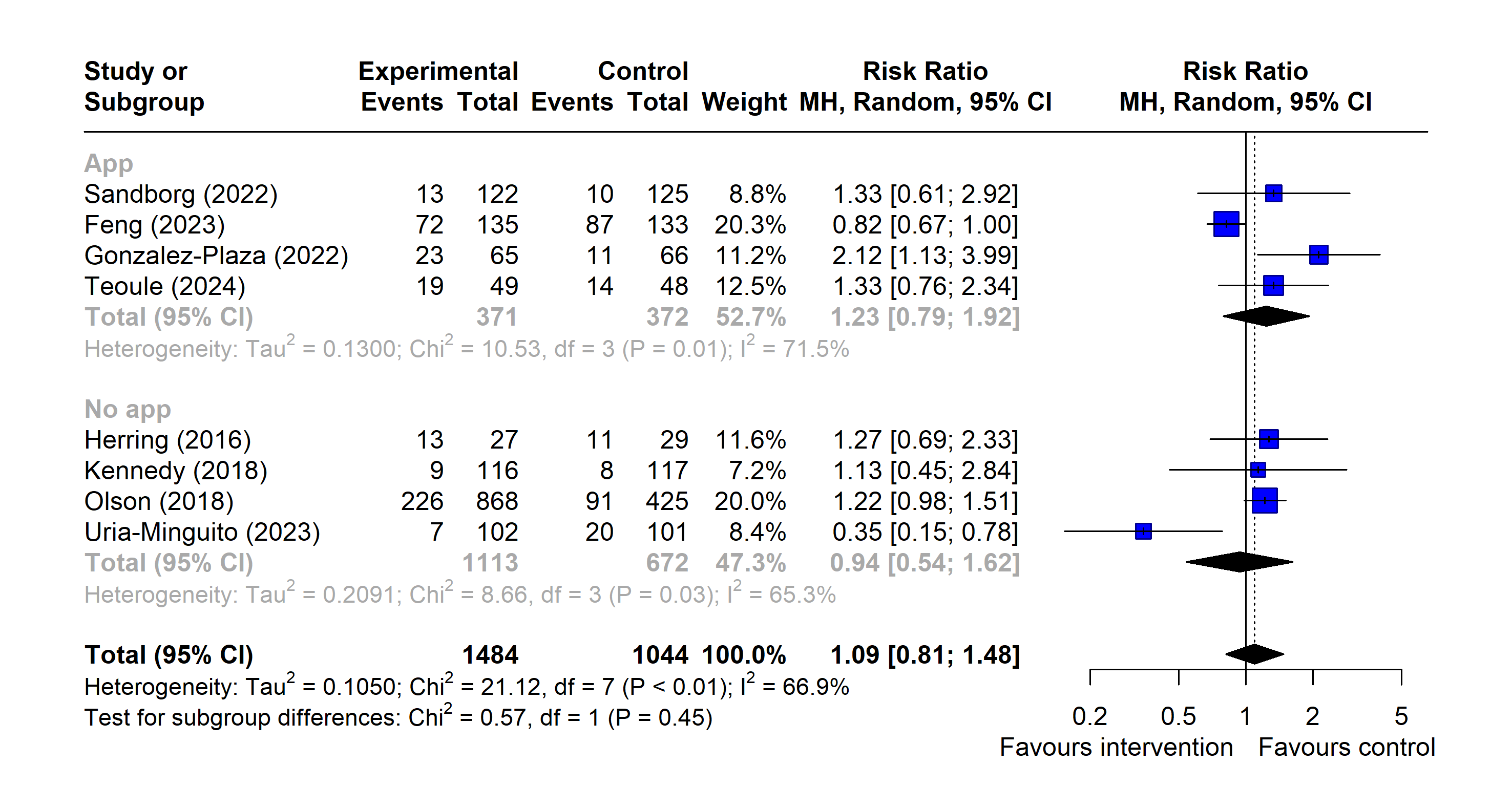
*

**Figure P*.*** Risk ratio for cesarean birth for digital health vs. usual care. The pooled effect is calculated by the Mantel–Haenszel random effects model. The subgroup is based on incorporating a mobile app in the digital health intervention.


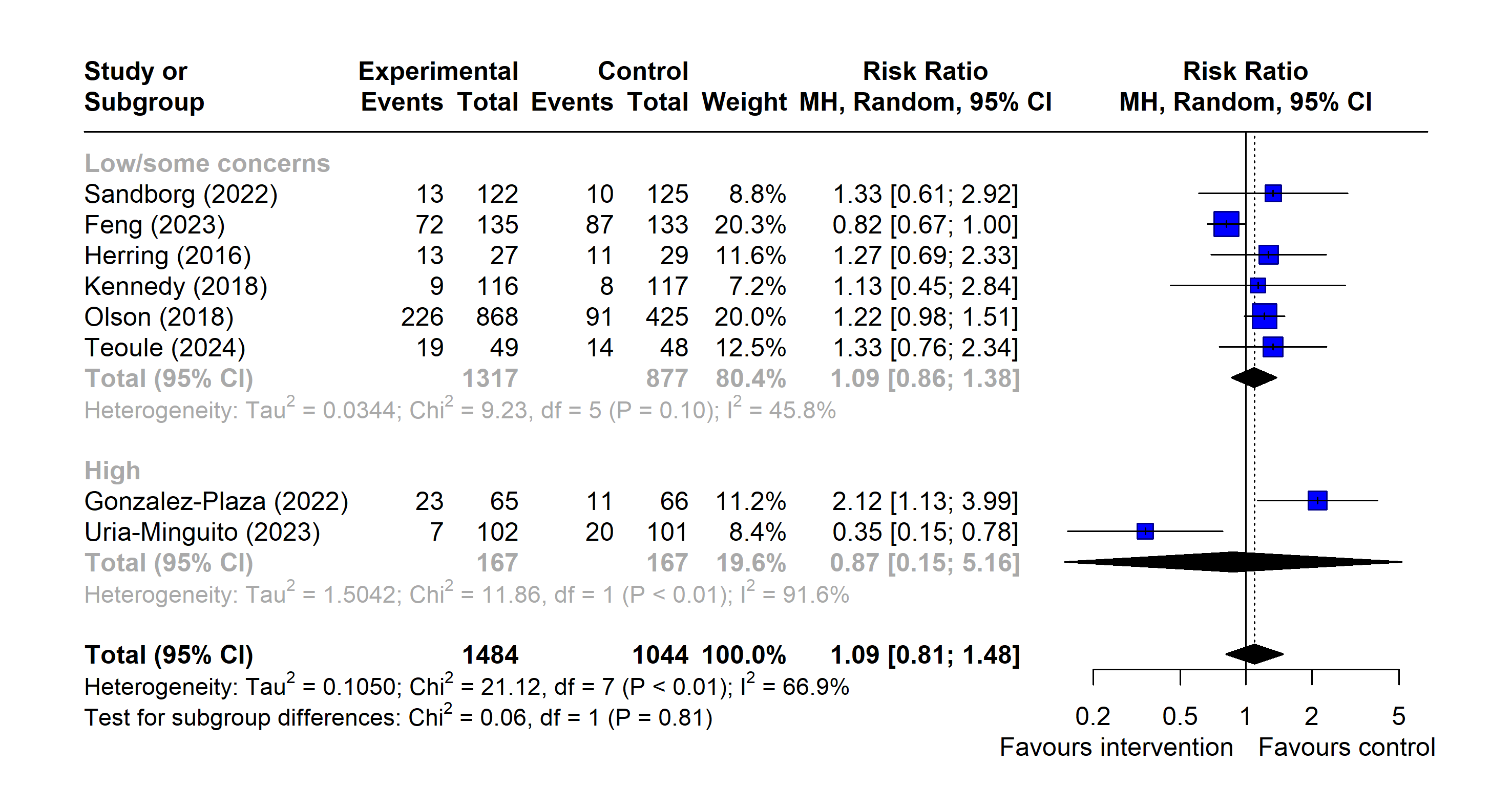


**Figure Q*.*** Risk ratio for cesarean birth for digital health vs. usual care. The pooled effect is calculated by the Mantel–Haenszel random effects model. The subgroup is based on the overall risk of bias of the study.


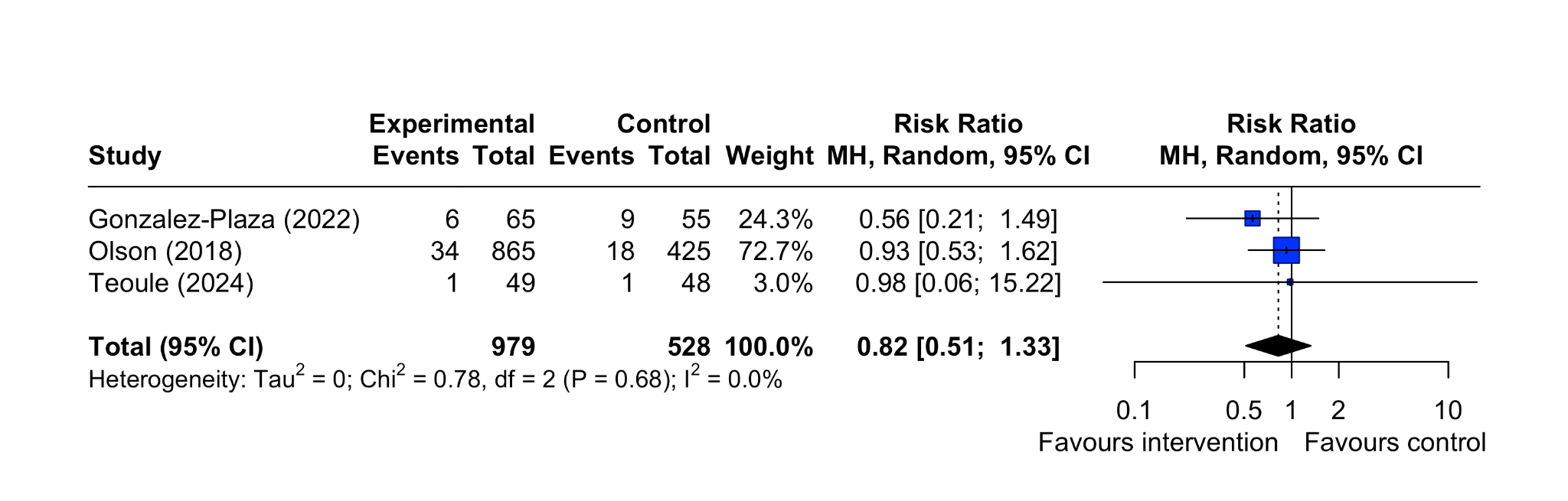


**Figure R*.*** Risk ratio for pre-eclampsia for digital health vs. usual care. The pooled effect is calculated by the Mantel–Haenszel random effects model.


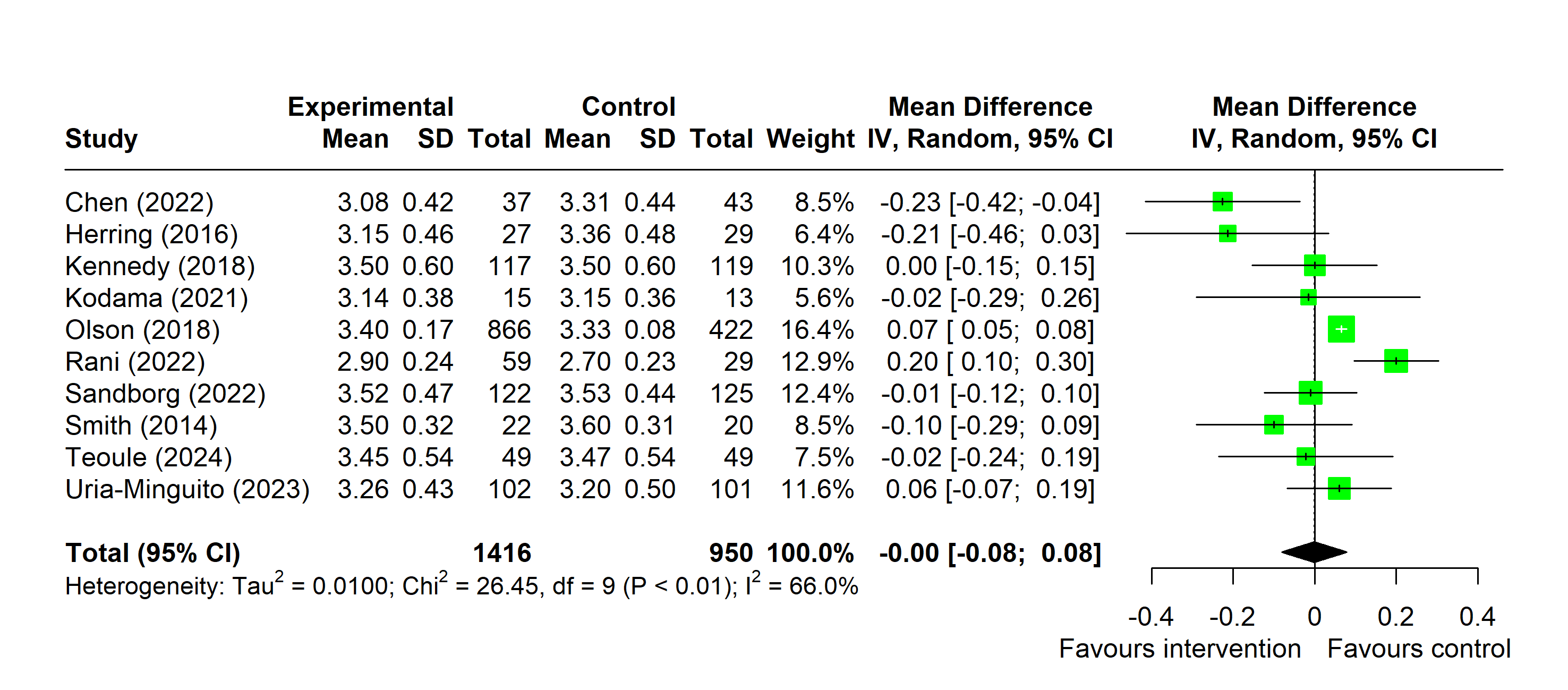


**Figure S.** Mean difference for birth weight (in kilogram) for digital health vs. usual care. The pooled effect is calculated by the DerSimonian-Laird random effects model.


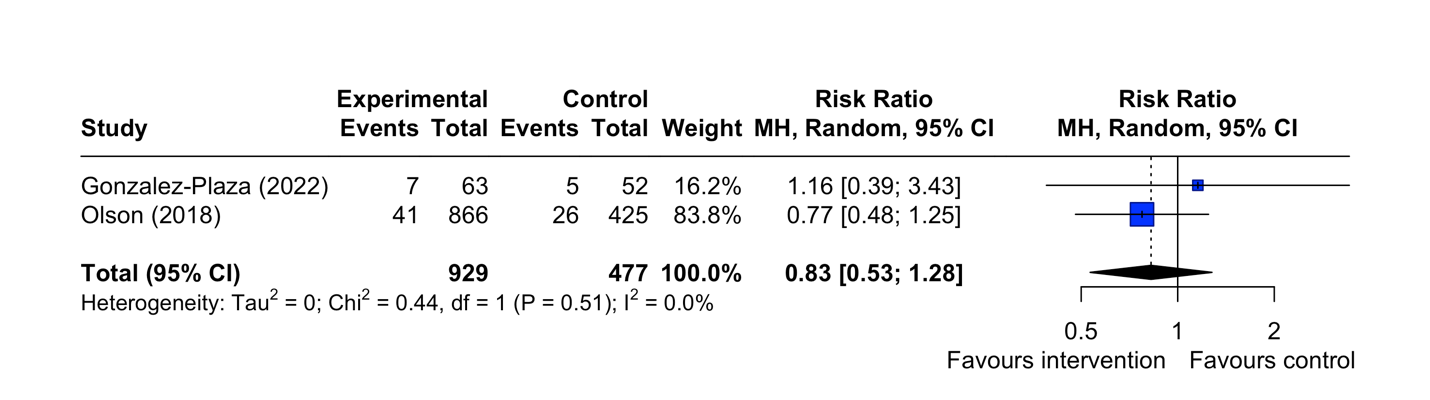


**Figure T*.*** Risk ratio for preterm birth for digital health vs. usual care. The pooled effect is calculated by the Mantel–Haenszel random effects model.
