# Supplementary material for: Effects of digital health counseling and behavioral interventions on weight management during pregnancy and postpartum: A systematic review and meta-analysis of randomized controlled trials
Source: PLoS One. 2025 Sep 25;20(9):e0331913. doi: 10.1371/journal.pone.0331913 (PMC12463243; doi:10.1371/journal.pone.0331913)
Supplement: S4 Appendix — (DOCX) [file pone.0331913.s004.docx]

**S4 Appendix.** Funnel plots.


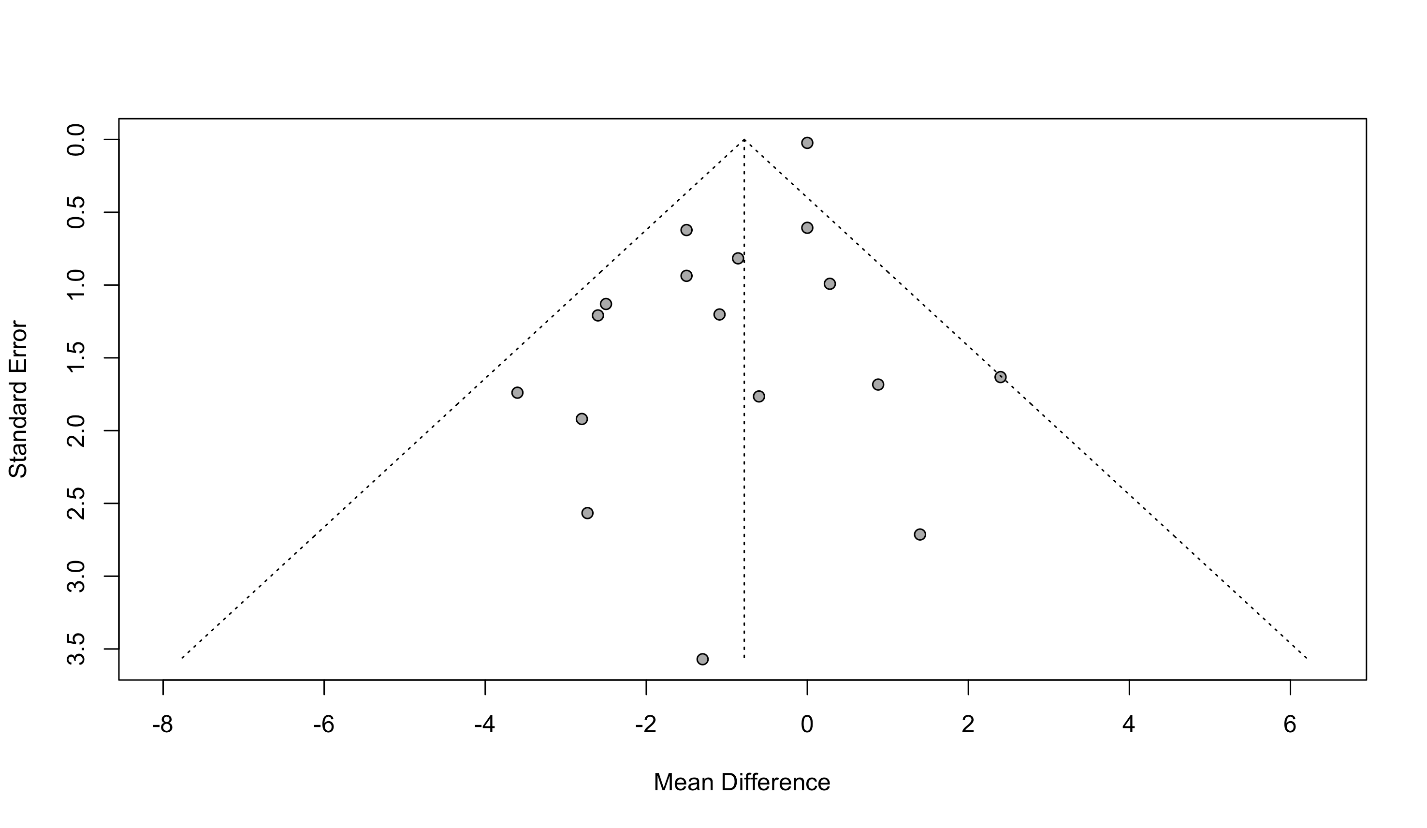


**Figure A*.*** Funnel plot for gestational weight gain outcome.


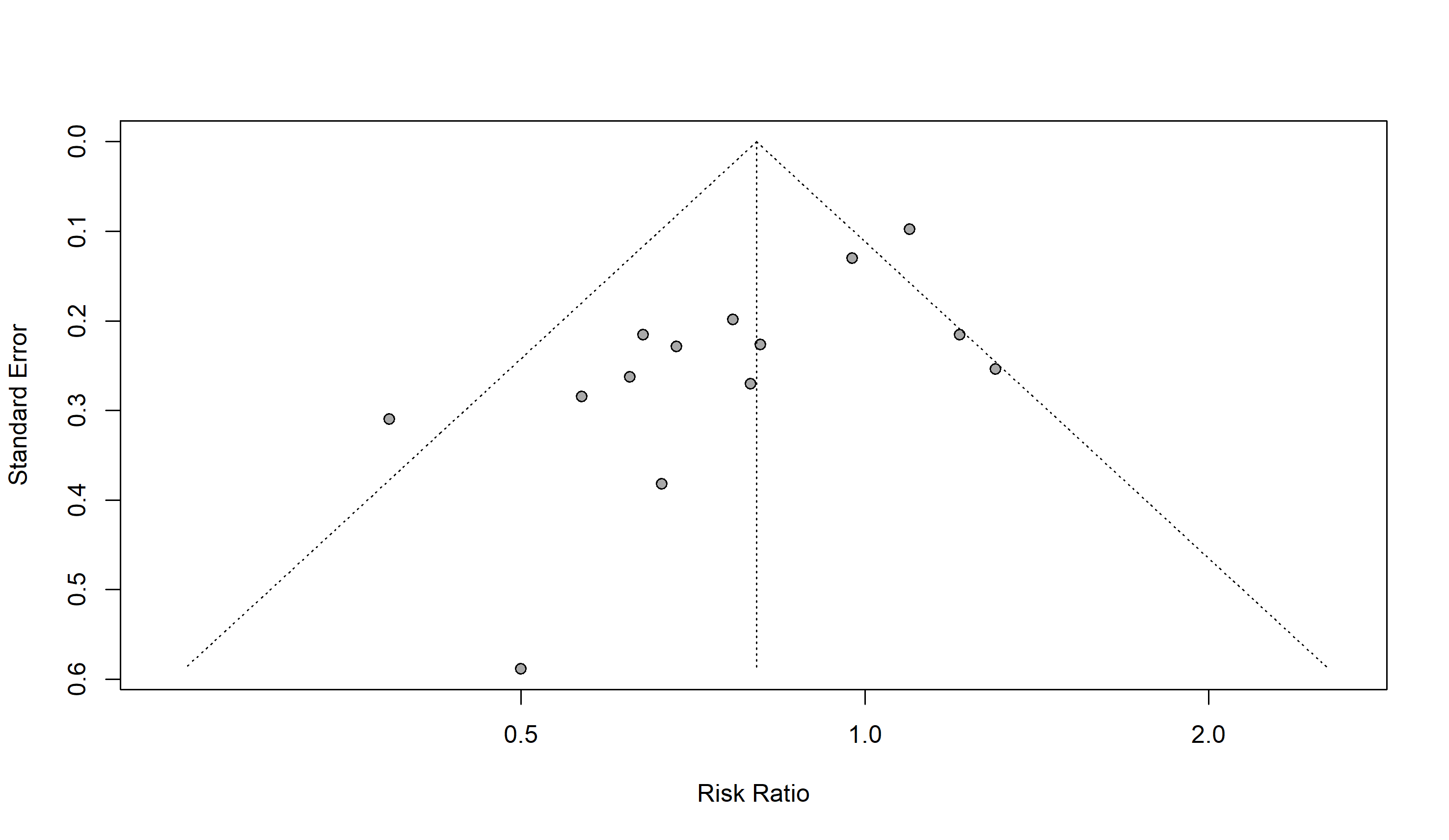


**Figure B*.*** Funnel plot for gestational weight gain exceeding IOM recommendations outcome.
