# Supplementary material for: Effects of digital health counseling and behavioral interventions on weight management during pregnancy and postpartum: A systematic review and meta-analysis of randomized controlled trials
Source: PLoS One. 2025 Sep 25;20(9):e0331913. doi: 10.1371/journal.pone.0331913 (PMC12463243; doi:10.1371/journal.pone.0331913)
Supplement: S1 Table — (DOCX) [file pone.0331913.s005.docx]

**S1 Table.** Characteristics of excluded studies and reasons for exclusion.

| **Author, year** | **Title** | **Reason for exclusion** |
| --- | --- | --- |
| Ainscough, 2016 | Impact of an mHealth supported healthy lifestyle intervention on behavioural stage of change in overweight and obese pregnancy | Abstract only |
| Singh, 2020 | Effects of Female Community Health Volunteer Capacity Building and Text Messaging Intervention on Gestational Weight gain and Hemoglobin Change Among Pregnant Women in Southern Nepal: A Cluster Randomized Controlled Trial | Not exclusively mHealth  Not weight related intervention |
| Uytsel, 2022 | Effect of the e-health supported INTER-ACT lifestyle intervention on postpartum weight retention and body composition, and associations with lifestyle behaviour: A randomized controlled trial | Not exclusively mHealth |
| Ainscough, 2018 | Impact of a smartphone app supporting a lifestyle intervention in overweight and obese pregnancy on maternal health and lifestyle outcomes | Not exclusively mHealth  No outcome of interest |
| Jackson, 2011 | Improving diet and exercise in pregnancy with Video Doctor Counseling: A randomized controlled trial | Intervention Less than 12 weeks |
| Tripette, 2014 | Home-Based Active Video Games to Promote Weight Loss during the Postpartum Period | Intervention Less than 12 weeks |
| Phelan, 2011 | Randomized trial of behavioural intervention to prevent excessive gestational weight gain: the Fit for Delivery Study | Not exclusively mHealth |
| Kennelly, 2018 | Pregnancy Exercise and Nutrition Support With Smartphone Application Support | Not exclusively mHealth |
| Ainscough, 2020 | Nutrition, Behaviour Change and Physical Activity Outcomes From the PEARS RCT - An mHealth-Supported, Lifestyle Intervention Among Preganant Women with Overweight and Obesity | Not exclusively mHealth |
| Senek, 2018 | An mHealth Walking Intervention for Pregnant Women with Obesity | Intervention Less than 12 weeks |
| Whooten, 2021 | Engaging fathers in the first 1000 days to improve perinatal outcomes and prevent obesity: rationale and design of the first heros randomized trial | Not exclusively mHealth |
| Deng, 2022 | Effects of Diet and Exercise Interventions to Prevent Gestational Diabetes Mellitus in Pregnant Women with High-Risk Factors in China: A Randomized Controlled Study | Not exclusively mHealth |
| Phelan, 2017 | Effect of an Internet-Based Program on Weight Loss for Low-Income Postpartum Women: A Randomized Controlled Trial | Not exclusively mHealth |
| Nobles, 2018 | The Effect of an Exercise Intervention on Gestational Weight Gain: The Behaviors Affecting Baby and You (B.A.B.Y.) Study: A Randomized Controlled Trial | Not exclusively mHealth |
| Phelan, 2014 | Does behavioral intervention in pregnancy reduce postpartum weight retention? Twelve-month outcomes of the Fit for Delivery randomized trial | Not exclusively mHealth |
| Yu, 2022 | Effects of 8-Week Online, Supervised High-Intensity Interval Training on the Parameters Related to the Anaerobic Threshold, Body Weight, and Body Composition during Pregnancy: A Randomized Controlled Trial | Not exclusively mHealth |
| Colleran, 2012 | Use of MyPyramid Menu Planner for Moms in a Weight-Loss Intervention during Lactation" | Not exclusively mHealth |
| Talebi, 2022 | Examination of influence of social media education through mobile phones on the change in physical activity and sedentary behavior in pregnant women: a randomized | Intervention Less than 12 weeks |
| Kennedy, 2016 | A pilot study: Women’s engagement with a nutrition, lifestyle and health website during pregnancy | No outcome of interest  Abstract only |
| Gallagher, 2024 | Acceptability of a novel text message delivered intervention for weight loss and maintenance of weight loss in the postpartum period: The Supporting Mums study | Abstract only |
| Huang, 2020 | Feasibility of conducting an early pregnancy diet and lifestyle e-health intervention: the Pregnancy Lifestyle Activity Nutrition (PLAN) project | Not exclusively mHealth |
| Okesene-Gafa, 2019 | Effect of antenatal dietary interventions in maternal obesity on pregnancy weight-gain and birthweight: Healthy Mums and Babies HUMBA) randomized trial | Not exclusively mHealth |
| Horn, 2018 | Dietary Approaches to Stop Hypertension Diet and Activity to Limit Gestational Weight: Maternal Offspring Metabolics Family Intervention Trial, a Technology Enhanced Randomized Trial | Not exclusively mHealth |
| Greene, 2018 | Do pregnant women with overweight and obesity find a nutrition and exercise intervention with smartphone app support acceptable? Findings from the PEARs randomised control trial | No outcome of interest |
| Ding, 2021 | WeChat‐assisted dietary and exercise intervention for prevention of gestational diabetes mellitus in overweight/obese pregnant women: a two‐arm randomized clinical trial | Not exclusively mHealth |
| Szmeja, 2014 | Use of a DVD to provide dietary and lifestyle information to pregnant women who are overweight or obese: a nested randomised trial | Not exclusively mHealth |
| Maturi, 2011 | Effect of physical activity intervention based on a pedometer on physical activity level and anthropometric measures after childbirth: a randomized controlled trial | Wrong population |
| Kim, 2022 | Psychological and Biochemical Effects of an Online Pilates Intervention in Pregnant Women during COVID-19: A Randomized Pilot Study | Intervention Less than 12 weeks |
| Evans, 2019 | Design of a novel digital intervention to promote healthy weight management among postpartum African American women | Wrong population  Protocol |
| Bennion, 2020 | Impact of an Internet-Based Lifestyle Intervention on Behavioral and Psychosocial Factors during Postpartum Weight Loss | Wrong outcome |
| Altazan, 2019 | Mood and quality of life changes in pregnancy and postpartum and the effect of a behavioral intervention targeting excess gestational weight gain in women with overweight and obesity: a parallel-arm randomized controlled pilot trial | Not exclusively mHealth |
| Lombard, 2016 | Preventing Weight Gain in Women in Rural Communities: A Cluster Randomised Controlled Trial | Not exclusively mHealth |
| McGirr, 2020 | Text messaging to help women with overweight or obesity lose weight after childbirth: the intervention adaptation and SMS feasibility RCT. | Wrong population |
| Nicklas, 2021 | Feasibility of an mhealth postpartum lifestyle intervention for women with cardiometabolic risk pre-and mid-covid: The fit after baby pilot randomized controlled trial | Abstract only |
| Wilcox, 2018 | A randomized controlled trial to prevent excessive gestational weight gain and promote postpartum weight loss in overweight and obese women: Health In Pregnancy and Postpartum (HIPP) | Not exclusively mHealth |
| Peaceman, 2017 | MOMFIT: A randomized clinical trial of an intervention to prevent excess gestational weight gain in overweight and obese women | Not exclusively mHealth   Abstract only |
| Chao, 2017 | A Pilot Randomized Controlled Trial of a Technology-Based Approach for Preventing Excess Weight Gain during Pregnancy among Women with Overweight | Abstract only (the full article included) |
